# A pathway toward clinical translation of hyperpolarized [1,4‐ ^13^C_2_ ,2,3‐d_2_]fumarate as an imaging biomarker for early cellular necrosis in vivo

**DOI:** 10.1002/mrm.30519

**Published:** 2025-05-07

**Authors:** Jonathan R. Birchall, Pascal Wodtke, Ashley Grimmer, Esben S. S. Hansen, Lotte B. Bertelsen, Nikolaj Bøgh, Marta Wylot, Maria J. Zamora‐Morales, Otso Arponen, Ines Horvat‐Menih, Elizabeth C. Latimer, Fung Tan, Evita Pappa, Johann Graggaber, Joseph Cheriyan, Kelly Holmes, Matthew J. Locke, Helen Sladen, Joan Boren, Mikko I. Kettunen, Anita Chhabra, Ian B. Wilkinson, Christoffer Laustsen, Kevin Brindle, Mary A. McLean, Ferdia A. Gallagher

**Affiliations:** ^1^ Department of Radiology University of Cambridge Cambridge UK; ^2^ Cancer Research UK Cambridge Centre Cambridge UK; ^3^ Department of Clinical Medicine Aarhus University MR Research Centre Aarhus Denmark; ^4^ Institute of Clinical Medicine University of Eastern Finland Kuopio Finland; ^5^ Radiopharmacy Department Cambridge University Hospitals NHS Foundation Trust Cambridge UK; ^6^ Division of Experimental Medicine and Immunotherapeutics, Department of Medicine University of Cambridge Cambridge UK; ^7^ Cambridge Clinical Trials Unit, Cambridge University Hospitals NHS Foundation Trust Cambridge UK; ^8^ The Discovery Centre, AstraZeneca, Cambridge Biomedical Campus Cambridge UK; ^9^ A.I. Virtanen Institute for Molecular Sciences, University of Eastern Finland Kuopio Finland; ^10^ Cancer Research UK Cambridge Institute Cambridge UK

**Keywords:** clinical translation, hyperpolarization, HP ^13^C‐MRI, imaging biomarker, necrosis, preclinical

## Abstract

**Purpose:**

The detection of hyperpolarized carbon‐13 (HP ^13^C)‐fumarate conversion to ^13^C‐malate using ^13^C‐MRSI is a biomarker for early detection of cellular necrosis. Here, we describe the translation of HP ^13^C‐fumarate as a novel human imaging agent, including the evaluation of biocompatibility and scaling up of the hyperpolarization methods for clinical use.

**Methods:**

Preclinical biological validation was undertaken in fumarate hydratase‐deficient murine tumor models and controls. Safety and biocompatibility of ^13^C‐fumarate was assessed in healthy rats (*N* = 18) and in healthy human volunteers (*N* = 9). The dissolution dynamic nuclear polarization process for human doses of HP ^13^C‐fumarate was optimized in phantoms. Finally, 2D ^13^C‐MRSI following injection of HP ^13^C‐fumarate was performed in an ischemia–reperfusion porcine kidney model (*N* = 6).

**Results:**

Fumarate‐to‐malate conversion was reduced by 42%–71% in the knockdown murine tumor model compared to wildtype tumors. Twice‐daily injection of ^13^C‐fumarate in healthy rats at the maximum evaluated dose (120 mg/kg/day) showed no significant persistent blood or tissue effects. Healthy human volunteers injected at the maximum dose (3.84 mg/kg) and injection rate (5 mL/s) showed no statistically significant changes in vital signs or blood measurements 1 h post‐injection. Spectroscopic evidence of fumarate‐to‐malate conversion was observed in the ischemic porcine kidney (0.96 mg/kg).

**Conclusion:**

HP ^13^C‐fumarate has shown promise as a novel and safe hyperpolarized agent for monitoring cellular necrosis. This work provides the basis for future imaging of HP ^13^C‐fumarate metabolism in humans.

## 1 INTRODUCTION

There is an unmet clinical need for the early detection of necrosis in diseases such as cancer, tissue ischemia, or infarction, and in metabolic disorders where early detection of cell death may aid the identification of successful response to therapy. For example, clinical assessment of tumor shrinkage may take several weeks or months to become detectable, but imaging metabolic changes may highlight this much earlier.^1^, ^2^ Hyperpolarized carbon‐13 MRI (HP ^13^C‐MRI) can facilitate the detection of tissue metabolism by increasing net nuclear spin magnetization by four to five orders of magnitude,^3^ enabling high SNR spectroscopic measurements and image contrast using a wide variety of probe molecules. Translating HP ^13^C‐MRI into the clinical setting could improve patient stratification, enable early detection of treatment response, and therefore reduce the burden on healthcare services in the future. However, the roadmap for biomarker translation from preclinical research to approval as a safe and reliable clinical decision‐making tool is challenging, particularly for intravenous contrast agents injected at high concentrations.^4^

The majority of clinical hyperpolarization studies have focused on [1‐^13^C]pyruvate for assessing glycolytic metabolism.^5^ However, because the labeled carbon at the C_1_ position does not enter the tricarboxylic acid cycle, as is the case for [2‐^13^C]pyruvate, this approach does not provide direct information on downstream metabolism.^6^ A range of promising additional molecules have been introduced preclinically,^7^ enabling pH imaging,^6^, ^8^, ^9^, ^10^ redox state assessment,^11^, ^12^, ^13^ and probing of metabolic pathways beyond the capabilities of pyruvate.^14^, ^15^, ^16^, ^17^

**FIGURE 1:**
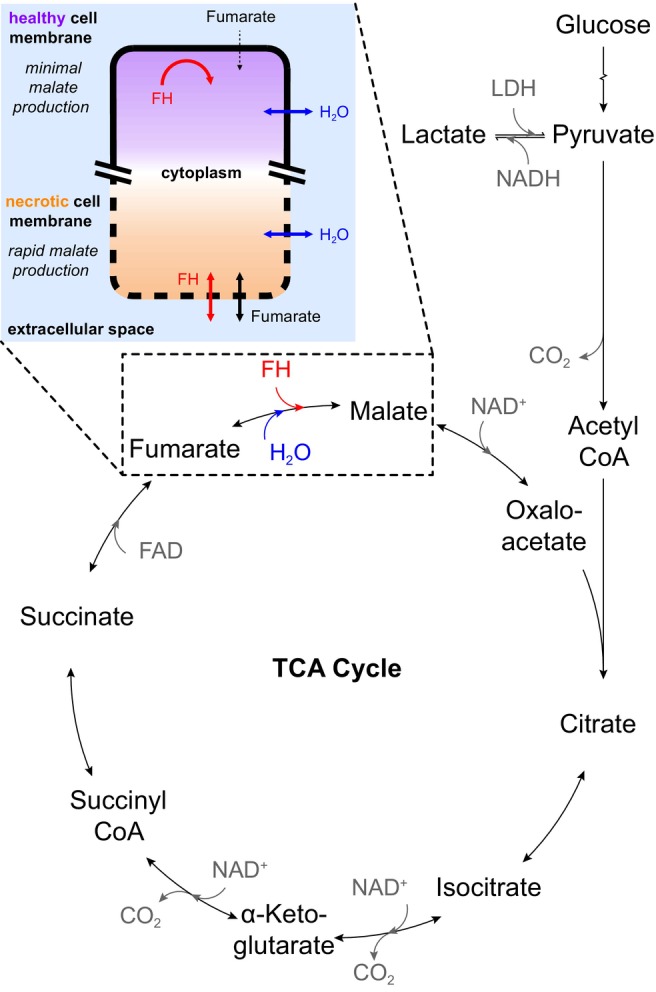
Fumarate‐to‐malate conversion via the enzyme FH (dashed box) within the TCA cycle, requiring only water as a cofactor for operation. Cross‐membrane leakage of fumarate and FH in necrosis is illustrated in the inset box, forming the basis of the proposed biomarker imaging. FH, fumarate hydratase; TCA, tricarboxylic acid.

Conversion of fumarate into malate via the enzyme fumarate hydratase (FH, also termed *fumarase* in less complex eukaryotes^18^) is a promising biomarker of cell death.^19^ Fumarate is taken up slowly from the extracellular space in healthy cells; however, following disruption of the cell membrane during cell death, FH is released into the extravascular space and extracellular fumarate rapidly enters the cell, facilitating metabolism.^17^ Importantly, the enzyme only requires water as a cofactor and therefore remains active in the context of cell death and outside the confines of the cell membrane, enabling a necrosis biomarker producing positive contrast for cell death (Figure 1).

Initial studies demonstrated increased HP ^13^C‐malate production in murine lymphomas following drug‐induced necrosis,^17^ with subsequent studies in hepatocellular carcinoma.^20^ The probe has been shown to monitor tumor shrinkage in response to chemotherapeutic agents in murine models of implanted human breast cancer^21^ and renal cell carcinoma,^22^ as well as for assessing vascular disrupting agents in murine lymphoma^23^ and xenograft models of colorectal cancer.^24^ In addition to oncological applications, the probe has been used to detect intramuscular necrosis^25^ and myocardial infarction^26^ in rats, acute kidney injury in mice,^27^, ^28^ and hypoxia‐mediated cell death in a rat model of diabetic nephropathy at an early stage prior to the onset of significant histological changes.^29^

This study outlines a pathway for the potential future clinical application of HP ^13^C‐fumarate‐to‐malate conversion as a biomarker for cellular necrosis in vivo, including the doses required to provide clinically useful information and associated safety aspects. We report on the toxicological and mutagenic safety of ^13^C‐fumarate in animals and describe the first injections in healthy human volunteers. Optimization of the ^13^C‐fumarate hyperpolarization process and signal acquisition on a clinical MRI system to support future human imaging studies is discussed. Lastly, we present 2D spectroscopic imaging of HP ^13^C‐fumarate‐to‐malate conversion in phantoms and an in vivo large animal model of ischemic reperfusion injury (IRI) to approximate human imaging. This work is of importance in the field of HP ^13^C‐MRI because very few molecules have crossed the translational gap into clinical use to date. In addition to ^13^C‐labeled pyruvate at the C_1_ and the C_2_ positions,^30^, ^31^, ^32^, ^33^, ^34^, ^35^ ^13^C‐labeled urea has been used as a biomarker for perfusion in prostate cancer,^36^ and [1‐^13^C]α‐ketoglutarate has been proposed as a probe for altered glutamate production in glioma.^37^ ^13^C‐fumarate has also now crossed the translational gap into humans.

## 2 METHODS

### 2.1 Evaluating HP fumarate‐to‐malate conversion in vivo using colorectal tumors after FH knockdown

FH knockdown was performed using short hairpin RNAs (shRNA) to characterize differences in HP ^13^C‐fumarate metabolism between FH knockdown tumors and controls. shRNA sequences were encoded in a DNA vector and introduced into a human colorectal model via plasmid transfection (LoVo, ATCC, Manassas, VA); these were compared to control tumors utilizing an empty vector. LoVo (ATCC) tumors have previously been shown to produce increased levels of malate following anti‐vascular endothelial growth factor (VEGF) treatment.^24^ Tumors were grown by implanting ˜5 × 10^6^ cells subcutaneously into the flanks of female C57BL/6 NOD‐SCID gamma mice (*N* = 7).

[1,4‐^13^C_2_]fumaric acid was dissolved in dimethyl sulfoxide (DMSO) containing a trityl radical (AH111501, GE HealthCare, Waukesha, WI) and was prepared using a dynamic nuclear polarization (DNP) hyperpolarizer (3.35 T, Oxford Instruments, Abingdon, UK). The sample was dissolved in a buffer containing 40 mM phosphate, 50 mM sodium chloride, and 40 mM sodium hydroxide at pH 7.4. The final injected concentration of HP ^13^C‐fumarate was approximately 20 mM (see Ref. 17 for detailed experimental methods).

^13^C‐MRS was performed using a 7 T horizontal bore magnet (Agilent Technologies, Santa Clara, CA) and an actively decoupled dual‐tuned proton (^1^H)/^13^C volume transmit coil with a 20 mm diameter ^13^C‐tuned surface receive coil (both Rapid Biomedical GmbH, Rimpar, Germany) placed over the tumor. Localization was determined using ^1^H spin‐echo imaging (see Ref. 38 for detailed experimental methods). For dynamic ^13^C‐MRS, a 6 mm oblique coronal slice through the tumor was chosen. Following this, 0.2 mL of HP ^13^C‐fumarate was injected intravenously into a tail vein over a period of 6 s, and the animal was immediately placed into the MRI scanner. Slice‐selective free induction decays were acquired with a 10° flip angle RF pulse, TR = 3 s with 60 repetitions. Spectra were analyzed in the time domain using a Java‐based MR user interface (jMRUI, http://www.jmrui.eu ^62^, ^63^), and signal amplitudes for [1,4‐^13^C_2_]fumarate and the combined signal from [1‐^13^C]malate and [4‐^13^C]malate were fitted.

Enzymatic activity of FH in tumors was estimated from the cumulative ratio of ^13^C‐malate/^13^C‐fumarate over time in both the FH knockdown (*N* = 4) and empty vector (*N* = 3) animals. After baseline imaging, animals received an intraperitoneal injection of bevacizumab (Avastin; Roche, Basel, Switzerland), an anti‐VEGF agent to induce tumor necrosis, at 5 mg/kg.^24^, ^39^ Animals were subsequently imaged at 48 and 72 h after treatment.

### 2.2 Toxicological studies in animals

Fumarate is an endogenous molecule and was therefore not expected to exhibit biological toxicity. However, because doses required for clinical imaging are supraphysiological, an assessment of toxicity was performed. All investigations were performed using double ^13^C‐labeled [1,4‐^13^C_2_,2,3‐d_2_]fumarate, with deuteration lengthening the spin lattice relaxation time and therefore enhancing the in vivo signal.^40^, ^41^

Ames and mouse lymphoma assay testing^42^ were undertaken (Covance Laboratories, Huntingdon, UK) to assess mutagenic potential (see section S1), along with analysis of in vitro hemocompatibility of ^13^C‐fumarate with rat blood in a 1.8% DMSO/0.08% AH111501/16.1% Trometamol buffer solution in water for injections to match the human formulation (see section S2). A repeated dose study with ^13^C‐fumarate in buffer solution was performed to assess systemic toxicity by monitoring changes in peripheral blood measurements and urinalysis following two doses (1 h apart), administered on days 1 and 8 to Sprague Dawley rats (*N* = 18). Potential medium‐term effects were assessed after a 2‐week recovery period. Animals were then culled to measure organ weights and note differences in macroscopic organ appearance and any histopathological changes. Platelet clumping and blood film assessments were undertaken to assess the effect of the ^13^C‐fumarate formulation on coagulation. The highest dose formulation assessed was 12 mg/mL, and the maximum dose volume for administration was 5 mL/kg twice daily. Therefore, the maximum dose assessed in this work was 60 mg/kg, representing a single exposure 15.6‐fold above the maximum human doses described below. Altogether, three male and three female rats were injected at each individual dose level of 5, 30, and 60 mg/kg. The ^13^C‐fumarate concentration in rat blood plasma was measured 5 min after each injection. Additional information on the toxicology study design, as well as additional details of blood and urinary parameters assessed and organs and tissues inspected at necropsy, are available in sections S3 and S4, respectively.

### 2.3 First‐in‐human safety and tolerability study

Nine healthy volunteers (6 male/3 female) received injections at the National Institute for Health Research (NIHR) Cambridge Clinical Research Facility. The first three volunteers received a low concentration (20 mM, 0.96 mg/kg) dose of ^13^C‐fumarate solution prepared under supervision of a pharmacist. Three volunteers then received a medium concentration (40 mM, 1.92 mg/kg) dose, with the final three receiving the maximum (80 mM, 3.84 mg/kg) dose. All volumes for injection were prepared as 0.4 mL/kg, and the three fumarate concentrations were injected at rates of 0.04, 0.4, and 5 mL/s. Participants were recruited and screened using blood tests; physical examination; medical history review; and monitoring of resting heart rate, temperature, and blood pressure. Favorable ethical approval for this study was awarded by East of England–Essex Research Ethics Committee and registered on a public website (REC: 20/EE/0090, IRAS number 266343).

At the start of each day of injection, a sample dose of [1,4‐^13^C_2_,2,3‐d_2_]fumarate (Sigma‐Aldrich, St. Louis, MO) was analyzed in a Good Clinical Laboratory Practice facility (Cambridge University Hospitals Clinical Investigation Ward, Cambridge, UK) to ensure the concentration was within specified limits (±10% tolerance of the target dose; see section S5). Filter integrity and sample pH tests were performed to ensure contrast agent sterility and safety for injection (target pH between 6.0 and 9.0). ^13^C‐fumarate was injected into the antecubital vein at the appropriate volume, flow rate, and dose concentration using a MedRad Spectris Solaris EP injection pump (Bayer AG, Leverkusen, Germany). After injection, participants were observed for a minimum of 1 hour while vital signs—temperature, blood pressure, heart rate, and oxygen saturation—were recorded. Blood samples were acquired before and after injection to assess safety parameters. Follow up by a clinician was performed within 72 h of injection, although a provision was in place for participants to contact a medical professional or study team member if required.

### 2.4 Hyperpolarization of [ ^13^C]fumarate at clinical doses

Dissolution DNP requires a glassing matrix and free radical^3^: DMSO (Wak‐Chemie Medical GmbH, Steinbach, Germany) and AH111501 trityl radical (Syncom B.V., Groningen, Netherlands), respectively, were used in this study. A dose of 0.38 g of clinical‐grade [1,4‐^13^C_2_,2,3‐d_2_]fumarate was first mixed with 0.66 g DMSO for 2 h at ˜30°C. Afterward, 19.1 mg of AH111501 was added, and the mixture was stirred for another 2 h at ˜30°C. The radical concentration was optimized (see below) for maximum polarization. The prepared sample was subsequently transferred into a cryovial and loaded into a 5 T SPINlab hyperpolarizer (GE HealthCare).

#### 2.4.1 Optimization of polarization

A pair of experiments were conducted in optimally hyperpolarized samples of ^13^C‐fumarate formulation identical to those mentioned above to optimize spin‐transfer efficiency. Microwave frequency was first incremented in steps of 1 MHz from 140.040 to 140.077 GHz (7.5 min acquisition time between frequencies). Then, microwave attenuation was varied over a range of 5 to 12 dB (step size 0.5 dB). Under each set of conditions, the steady‐state HP ^13^C‐fumarate signal was recorded. The maximum achievable HP ^13^C‐fumarate signal was assessed at steady state following microwave irradiation at 140.055 GHz (chosen based on the optimal frequency for pyruvate on our system; see section S6) and with 8 dB attenuation in the SPINlab hyperpolarizer at 0.8 K using different concentrations of AH111501 (17.5, 20, 25, and 30 mM). An otherwise fixed formulation of 0.38 g fumarate and 0.66 g DMSO with varying radical content (16.7, 19.1, 23.9, and 28.7 mg) was used throughout.

#### 2.4.2 Dissolution

Hyperpolarized samples were dissolved in 51 mL of superheated (>100°C) and pressurized sterile water. In the acidic fluid, AH111501 precipitated and was filtered to reduce the final injected radical content to <10 μM. Subsequently, the sample was neutralized by a sterile buffer solution (Royal Free London NHS Foundation Trust, Pharmaceutical Quality Control Laboratory, London, UK; manufactured under Specials Manufacturing Licence MS11149) containing sterile water (25.5 mL), sodium hydroxide (11.35 g), and tromethamine (TRIS, 7.25 mL); pH = 13.4. The sample was passed through a sterile filter with a pore size of 0.22 μm and was drawn into a MedRad syringe (Bayer AG) while an aliquot (˜5 mL) was used for quality control prior to injection. The time for dissolution and sample ejection was ˜30 s.

#### 2.4.3 Quality control (QC)

A multi‐probe QC unit was used to measure solution temperature as well as fumarate and AH111501 concentration prior to injection. Sample pH was measured spectrophotometrically using a calibration file tailored for fumarate. QC was performed on a similar timescale as dissolution and ejection from the hyperpolarizer and was conducted simultaneously with sample transfer to either a 50‐mL Falcon tube (for phantom experiments) or a MedRad syringe pump (Bayer AG) for injection in vivo. Therefore, the sample was ready for injection after approximately 60 s.

### 2.5 Phantom studies

HP ^13^C‐fumarate imaging in phantoms was performed using a 26 cm inner diameter dual‐tuned ^1^H/^13^C transmit/receive head coil (Rapid Biomedical GmbH) at ˜32.1 MHz and a field strength of 3 T (MR750, GE HealthCare). Unlocalized MRS applying 18 sequential scans of 32 μs pulse length was used to observe longitudinal relaxation of ^13^C nuclear spins at a TR of 10 s and over a 5 kHz bandwidth. *T*_1_ estimates were not corrected for RF losses but were assumed to be negligible due to the low nominal flip angle used (3°). This was followed by a hard pulse 2D MRSI acquisition: FOV = 20 cm, TR = 116 ms, TE = 0.237 ms, pulse width = 100 μs, flip angle = 10°, 10 × 10 weighted circular k‐space coverage with 61 transients, 5 kHz bandwidth, 7 s total duration. Dotarem (Guerbet, Villepinte, France) was then added to the ^13^C‐fumarate solution as a source of gadolinium contrast agent in a 1:200 ratio, and the Falcon tube was inverted several times to mix. This served to increase the spin relaxation rate and enable calculation of thermal ^13^C polarization using 12 transients at a 90° flip angle and 500 μs pulse length each, with a TR of 1 s as described in section S8.

Lastly, a 3D fast spoiled gradient echo ^1^H pulse sequence (FOV = 20 cm, TR = 6.2 ms, TE = 1.88 ms, 64 locations, flip angle = 5°, 2 averages, 42 s total duration) was acquired to facilitate coregistration of HP ^13^C images with structural ^1^H MRI.

Spectroscopic imaging of FH activity at differing enzyme concentrations was also performed. Upon dissolution of the HP ^13^C‐fumarate solution, 15 mL was rapidly transferred into three separate 50‐mL Falcon tubes, each containing either 0, 50, or 100 unit quantities of FH (Sigma‐Aldrich), chosen to cover a wide range of physiological FH concentrations from normal tissue to tumor cells as reported previously.^17^, ^23^, ^43^ Tubes were inverted three times to ensure proper mixing prior to transfer into the MRI scanner, where an unlocalized MRS sequence (described above) was run every 10 s after insertion. Fumarate‐to‐malate conversion was assessed with the 2D dynamic MRSI sequence described previously. Peak identification and integration over each voxel were performed using MatLab (version 2023b, MathWorks, Natick, MA), and intensity ratios of HP fumarate (single peak) and malate (two peaks, arising from the inequivalent ^13^C_1_ and ^13^C_4_ resonances) were compared to produce spatial maps of the malate/fumarate ratio in each phantom after ˜6 min of metabolism. Spectrophotometric analysis at 290 nm was used to confirm these findings (see section S9).

### 2.6 Large animal studies

For the porcine IRI experiments, hyperpolarization was performed using a SpinAligner DNP hyperpolarizer (Polarize, Frederiksberg, Denmark)^44^ operating at 6.7 T and 1.4 K. The vial content for hyperpolarization was 0.38 g [1,4‐^13^C_2_,2,3‐d_2_]fumarate/0.66 g DMSO/34.3 mg AH111501 (36 mM radical concentration after dissolution). Injection was performed following dilution in 40 mL water for injection at a rate of 5 mL/s, followed by a 20 mL saline flush. Fumarate concentration was measured using a SpinSolve benchtop NMR spectrometer (Magritek GmbH, Aachen, Germany), comparing signal intensity against a linear fit of [1,4‐^13^C_2_,2,3‐d_2_]fumarate titrations at five different concentrations.

Imaging was performed 20 s after ^13^C‐fumarate injection on a 3 T MRI scanner (Signa, GE Healthcare) using a single 20 mm slice CSI sequence (FOV = 28 cm, TR = 115 ms, TE = 2.71 ms, flip angle = 10°, 20 × 20 mm voxel size, 196 transients, 5 kHz bandwidth, 23 s total duration). A combination of clamshell transmit (Rapid Biomedical GmbH) and eight‐channel flexible receiver array coil (JD Coils, Hamburg, Germany) were employed. Zero‐filling was performed during reconstruction to double the 2D MRSI image resolution from 20 × 20 × 20 mm^3^ (14‐by‐14 voxels) to 10 × 10 × 20 mm^3^ (28‐by‐28 voxels). Phase correction and peak fitting of fumarate and malate spectra were performed using the Oxford Spectroscopy Analysis toolbox for MatLab (version 2023b, MathWorks).^45^

This study was undertaken in six pigs (all female, weight = 40 ± 2 kg) as approved by the Danish Animal Inspectorate (Ref. 2019‐15‐0201‐00367). In each animal, the right kidney underwent 90 min of ischemia and subsequent warm IRI^46^ (see Ref. 47 for detailed experimental methods), and then was imaged after 3–4 h by intravenous injection of HP ^13^C‐fumarate. No IRI was induced in the contralateral (left) kidney of each animal serving as a noninjured control.

## 3 RESULTS

### 3.1 Measurements of metabolism in a FH knockdown tumor model

**FIGURE 2:**
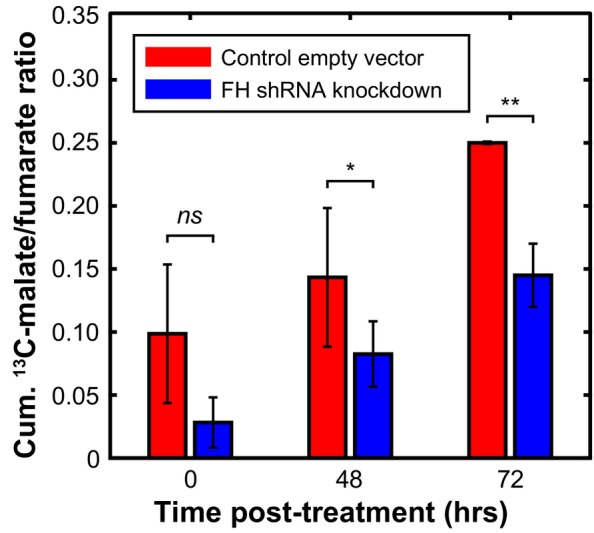
Comparison of the cumulative ^13^C‐malate/^13^C‐fumarate ratio over time following treatment with bevacizumab in control tumors (red, empty vector) and FH knockdown tumors (blue, shRNA for FH). ^13^C, carbon‐13; FH, shRNA, short hairpin RNA.

Figure 2 shows results from the in vivo tumor experiments. In control tumors (empty vector) where FH enzyme was present, a higher ^13^C‐malate/^13^C‐fumarate ratio was observed across all timepoints compared to knockdown tumors, indicating physiological conversion of fumarate to malate within the tumor. In the FH knockdown tumors, there was between 42% and 71% reduction in the ^13^C‐malate/^13^C‐fumarate ratio across all timepoints. A progressive increase in this ratio after 48 and 72 h of anti‐VEGF therapy with bevacizumab indicated increasing necrosis over this period. The differences in ^13^C‐malate/^13^C‐fumarate at 48 and 72 h post‐therapy were statistically significant as determined via a two‐sample *t*‐test with equal variance (*p* = 0.037 and 0.003, respectively).

### 3.2 Fumarate safety and toxicology

#### 3.2.1 Preclinical assessment of toxicology

Ames and mouse lymphoma assay testing showed no evidence of mutagenic activity (see Table S1). Platelet clumping assessments and blood film reviews showed no evidence of coagulation. Statistical analysis of in vitro hemocompatibility with rat blood suggested that injections at a fumarate formulation/blood ratio of 1.35:1 may affect some coagulation parameters, including activated partial thromboplastin clotting time, prothrombin time, and fibrinogen concentrates (see Table S2). However, there was no evidence of hemolysis, suggesting that although the buffer solution may affect prothrombin time, the fumaric acid formulations had little effect on hemocompatibility. Elongated clotting times observed at a formulation/blood ratio of 2.70:1 were likely due to the large resultant dilution factor. Platelet clumping assessments, blood film reviews, and hemolysis assessments showed no evidence of hemotoxicity.

whilst [1,4‐^13^C_2_,2,3‐d_2_]fumarate was well tolerated, slight physiological changes were observed, including elevated white blood cell count, increased group mean alkaline phosphatase activity, elevated plasma phosphorus concentrations, and higher organ weights at doses of 60 or 120 mg/kg/day. Additionally, minimal diffuse cortical vacuolation in the zona reticularis was seen in female rats only at very high doses of 120 mg/kg/day. This dose was 15.6‐fold higher than the highest injected human dose and was therefore not deemed to be clinically relevant. Full recovery of these findings was observed after the 2‐week recovery period. The no‐observed‐adverse‐effect level, the experimental dose level immediately below that which produced a statistically significant increase in the rate of adverse effects observed relative to healthy control animals,^48^ was 120 mg/kg/day for both sexes in this study. More information on significant toxicity and pathological observations can be found in Tables S5–S11.

**FIGURE 3:**
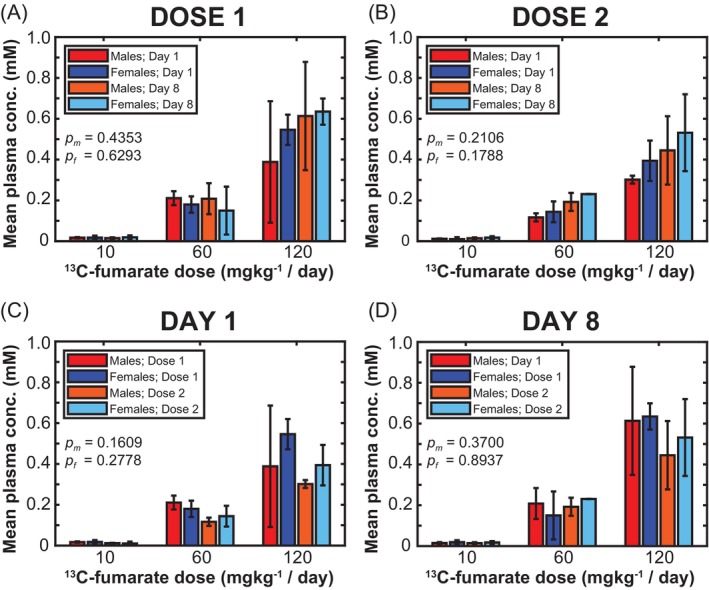
Mean rat blood plasma concentrations of ^13^C‐fumarate following administration of different doses of ^13^C‐fumarate in solution after the: (A) first dose on days 1 and 8, (B) second dose on days 1 and 8, (C) first and second doses on the first day, (D) first and second doses on day 8. Error bars are included as the SD of the population average. The *t*‐test *p*‐values are quoted separately for male rats (*p*_*m*_) and female rats (*p*_*f*_).

Results from the repeated dose toxicity study are illustrated in Figure 3. A series of paired, two‐tailed t‐tests determined that no statistical significance (0.16 ≤ *p* ≤ 0.89) could be confirmed between ^13^C‐fumarate concentration in the blood plasma and the size of the ^13^C‐fumarate solution dose over the 7 day period (Figure 3A,B), or over the 1 h period between doses (Figure 3C,D). This indicated rapid metabolism and/or excretion of ^13^C‐fumarate with no significant accumulation in blood over time.

#### 3.2.2 First‐in‐human healthy volunteer injections

**FIGURE 4:**
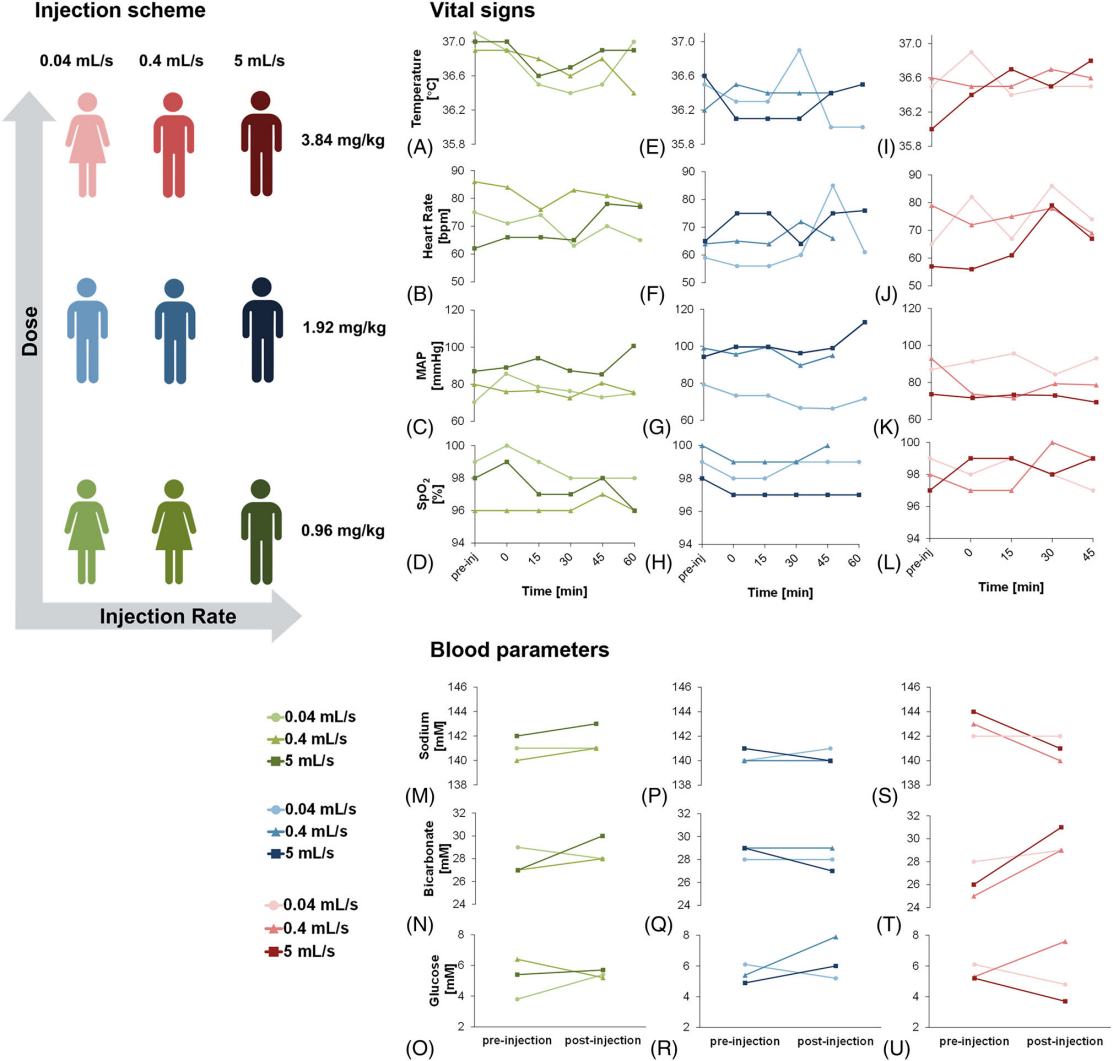
Participant vital measurements before and after injection with ^13^C‐fumarate: (A–D) 0.96 mg/kg, (E–H) 1.92 mg/kg, (I–L) 3.84 mg/kg. Blood biochemistry measurements before and after injection of ^13^C‐fumarate, (M–O) 0.96 mg/kg, (P–R) 1.92 mg/kg, (S–U) 3.84 mg/kg. Injection rates can be identified by line color throughout (see injection scheme in the top‐left of the image).

Figure 4 displays the recorded participant temperature, heart rate, blood pressure, and oxygenation, as well as blood biochemistry including sodium, bicarbonate, and glucose levels before and after injection as a function of ^13^C‐fumarate dose and injection rate. All nine injections were within the acceptable pH range (min 7.23, max 8.29). Additional parameters assessed are included in Table S12 and Figure S1.

No substantial changes in participant temperature, heart rate, blood pressure, or oxygenation were recorded over the 2 h post‐injection. Comparison of blood samples acquired from each participant before and after ^13^C‐fumarate injection also demonstrated no statistically significant changes. However, a >10% increase in the quantity of unlabeled bicarbonate was observed in two of the nine participants (14% and 16%; see Figure 4T). Participants were otherwise clinically asymptomatic with no adverse events reported.

### 3.3 Optimization of [ ^13^C]fumarate polarization

**FIGURE 5:**
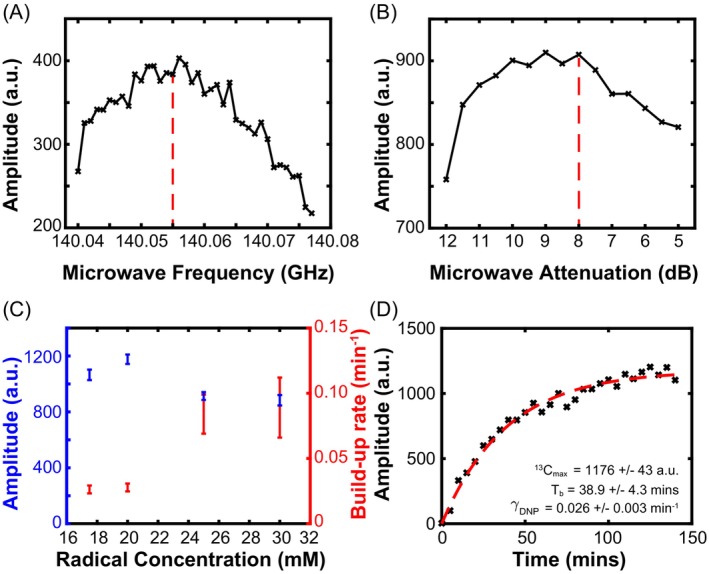
Optimization of microwave (A) frequency and (B) attenuation for hyperpolarization of ^13^C‐fumarate on the SPINlab hyperpolarizer (GE HealthCare, Waukesha, WI). Vertical dashed red lines indicate chosen optimal values utilized in later experiments. (C) Steady‐state ^13^C polarization and ^13^C polarization buildup rate of HP ^13^C‐fumarate in the presence of various AH111501 radical concentrations. (D) Example ^13^C polarization buildup over time (20 mM radical variant pictured; other concentrations shown in Figure S3). HP, hyperpolarized.

Peak signal intensity was recorded at 140.055 GHz (Figure 5A). Optimal signal amplitude and SNR were achieved at a microwave attenuation level of 8 dB (Figure 5B). Results from varying the radical concentration are shown in Figure 5C. An example timecourse of ^13^C polarization buildup using optimal conditions is shown in Figure 5D: Hyperpolarization buildup curves at each radical concentration are shown in Figure S2. Comparable sweeps of signal intensity as a function of microwave frequency and attenuation for [1‐^13^C]pyruvate are presented in Figure S3.

At these optimized settings, a polarization of ˜19% was recorded from the sample containing 20 mM AH111501 radical, with an associated polarization buildup rate of *γ* = 0.026 ± 0.003 min^−1^. Significantly elevated buildup rates were observed at higher radical concentrations, and these were accompanied by only marginal losses in the maximum polarization achieved: ˜14% at *γ* = 0.089 ± 0.023 min^−1^ when utilizing the 30 mM radical concentration.

### 3.4 Phantom imaging

**FIGURE 6:**
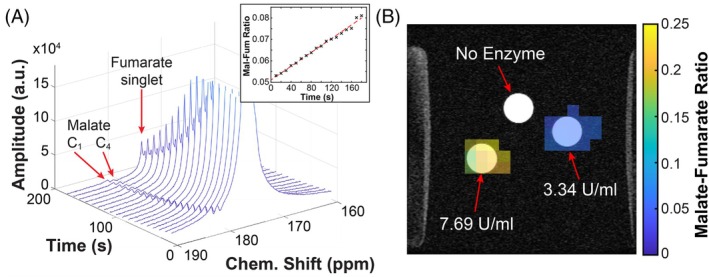
(A) ^13^C MR spectra of HP fumarate and malate over time in the ˜7.7 U/mL FH‐containing phantom (corresponding malate/fumarate ratio plot inset). The truncated signal from the HP [1,4‐^13^C_2_,2,3‐d_2_]fumarate is at 176.0 ppm, the signal from [1‐^13^C]malate at 182.5 ppm, and the signal from [4‐^13^C]malate at 181.4 ppm. (B) ^1^H structural image overlaid with a 2D colormap of HP malate/fumarate ratio per voxel in each of the three phantoms acquired after ˜6 min of enzyme activity, assessed by peak‐area integration of the respective MRSI spectra.

Dynamic MR spectroscopic measurements of HP fumarate and malate signals observed in the 50‐mL Falcon tube phantom containing the highest FH concentration are shown in Figure 6A. The *T*_1_ of HP ^13^C‐fumarate was calculated via exponential fitting to be ˜76 s, with magnetization decaying to around 45% of its initial solid‐state polarization during the ˜60 s dissolution and sample transfer time. The insert shows a roughly linear increase in malate/fumarate ratio over time, as determined by area‐under‐curve integration at each timepoint. Figure 6B demonstrates quantitative MRSI measurement of the malate/fumarate ratio in three otherwise identical phantoms containing HP ^13^C‐fumarate solution and different quantities of FH. Spectrophotometric characterization measuring the fumarate‐to‐malate conversion in a ˜5 U/mL enzyme‐containing phantom over time can be found in Figure S4.

### 3.5 In vivo imaging of renal ischemia in a porcine model

**FIGURE 7:**
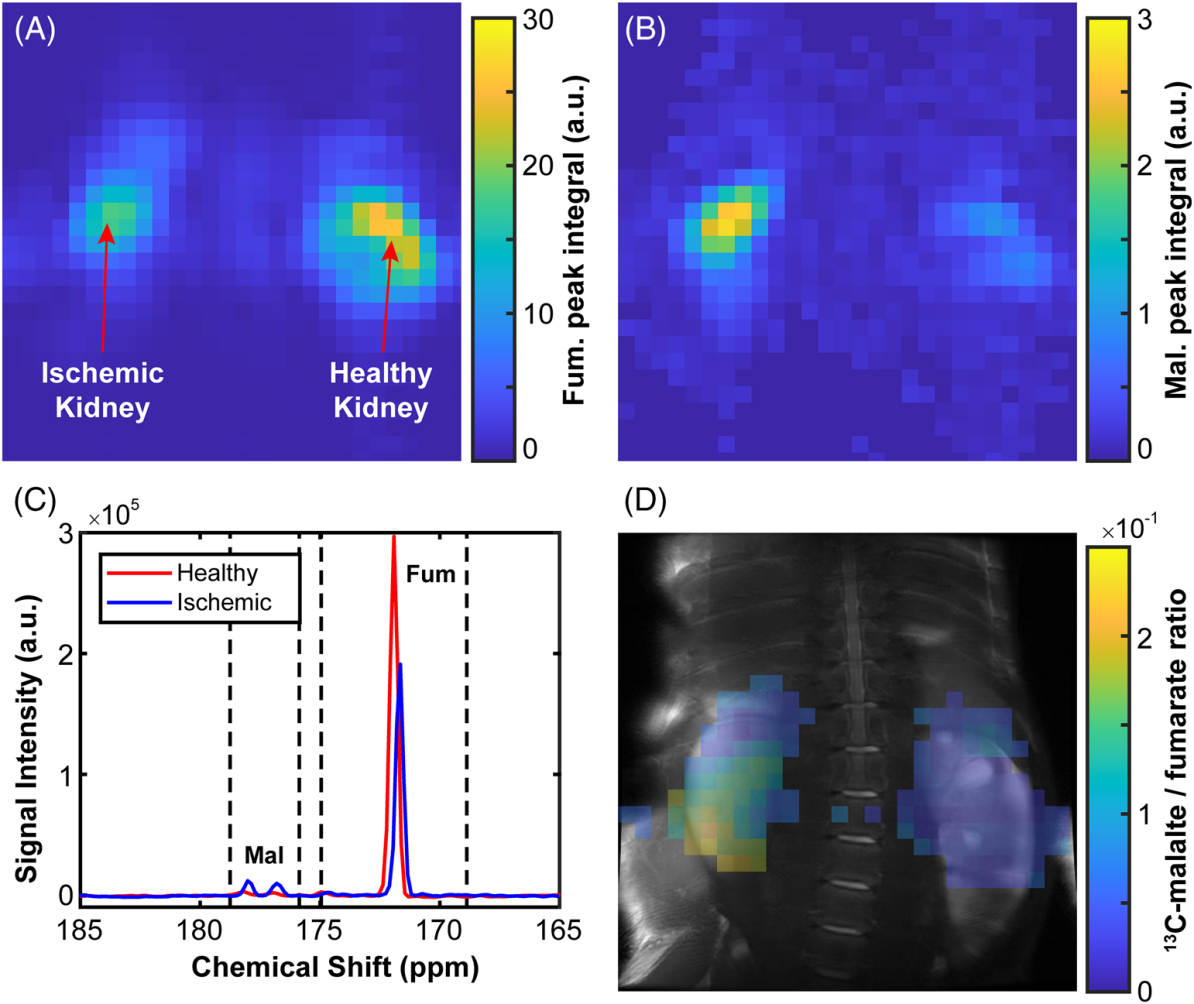
Spatial colormaps showing the peak area integral by voxel for the: (A) ^13^C‐fumarate peak, and (B) two ^13^C‐malate peaks from 2D MRSI acquisition. (C) Example MR spectra of HP ^13^C‐fumarate and ^13^C‐malate in the ischemic right kidney (blue line) and contralateral healthy kidney (red line), 20 s after injection. The integration ranges for the respective spectra are indicated by dashed vertical lines. (D) Spatial colormap of the ratio of malate‐to‐fumarate peak area integrals outlined in displays (A, B), coregistered with the ^1^H MRI localizer.

HP fumarate and malate peak integrals were measured across all voxels during the 2D MRSI acquisition to produce spatial colormaps of the individual fumarate and malate signal intensities per voxel (Figure 7A,B). Example HP ^13^C‐fumarate and ^13^C‐malate spectra obtained from single voxels are shown in Figure 7C. HP ^13^C‐malate peaks were visible only in the ischemic kidney, at approximately +6 ppm relative to the corresponding HP ^13^C‐fumarate peak. The malate/fumarate ratios per voxel across both the ischemic and contralateral normal kidney are shown in Figure 7D, overlaid on an anatomical ^1^H image. Polarization at the point of dissolution was estimated to be ˜27%. A histogram plot of this data comparing the ischemic and healthy kidneys, along with corresponding data from other animals imaged in the study, are shown in Figure S5.

In the example presented, an elevated ^13^C‐malate/^13^C‐fumarate ratio was observed in the ischemic porcine kidney relative to the healthy control: 0.08 (*σ* = 0.05) compared to 0.03 (*σ* = 0.02) for all voxels considered above the noise threshold (i.e., those visible in Figure 7D).

## 4 DISCUSSION

The results demonstrate the feasibility of using HP ^13^C‐fumarate as a clinical probe for imaging cellular necrosis using MRI. We have optimized sample preparation for clinical use and demonstrated the safety of the probe preclinically and in healthy human volunteers. Furthermore, we have shown that knocking down FH in a murine tumor model results in a reduction in the ^13^C‐malate/^13^C‐fumarate ratio, confirming that the method measures enzyme activity within tissue. Finally, when utilizing the clinical dose preparation of HP ^13^C‐fumarate in a large animal model, ^13^C‐malate signal was detected following ischemia–reperfusion injury, demonstrating the potential of this approach for detecting necrosis in patients in the future.

The in vivo tumor experiments in mice showed a two‐ to threefold reduction in the ^13^C‐malate/^13^C‐fumarate ratio following knockdown of FH expression using shRNA. The smaller reduction in enzyme activity in vivo compared to in vitro following shRNA knockdown likely reflects the contributions from cells within the tumor microenvironment that have normal FH expression, such as the stroma, immune cells, or vascular tissue. Alternatively, there may be some restoration of FH activity in tumor cells due to clonal expansion of populations with higher FH expression. The malate signal increased following the administration of an antivascular agent in keeping with previous work,^24^ but this remained significantly lower in the knockdown tumors compared to the control tumors over the three timepoints. These results show that HP ^13^C‐fumarate can be used to measure tumor FH activity and changes with the onset of cell death—and also potential complexity of the biological origin of these measurements in that multiple tissue compartments may contribute to the detected signal.

Injections into healthy volunteers showed no significant physiological changes, confirming the safety of fumarate as an endogenous molecule. The >10% increase in bicarbonate concentration in two of the participants may have arisen from the pH of the injected probe being at the upper end of the acceptable range in these cases (pH: 8.29, 8.20), resulting in a transient change in the physiological bicarbonate/carbon dioxide ratio to compensate by buffering these changes.

The maximum achievable HP ^13^C signal was obtained using a radical concentration of 20 mM. However, the more rapid polarization buildup rate recorded using a radical concentration of 30 mM (˜3.4‐fold higher) may be more desirable for scaling up contrast agent production in a future clinical setting given the significant reduction in sample polarization time. Previous characterization of [1‐^13^C]pyruvate hyperpolarization indicated an optimal concentration of ˜25 mM AH111501,^49^ which is in reasonable agreement with our results. The observation that the polarization buildup was efficient for both ^13^C‐fumarate and ^13^C‐pyruvate at identical microwave frequency and power implies the potential for co‐polarization and sequential injection of these contrast agents to target different metabolic processes.^50^ Simultaneous injection has been demonstrated for HP ^13^C‐labeled pyruvate and urea,^20^, ^25^, ^36^, ^51^ although spatially resolving malate and pyruvate–hydrate peaks may be challenging at clinical field strengths in the case of ^13^C‐labeled pyruvate and fumarate co‐injection.^52^

The presence of increased HP ^13^C‐malate signal in the ischemic pig kidney (Figure 7B,C) is likely to be as a result of either increased FH activity in the extracellular space or increased permeability of the membrane to fumarate following acute cellular necrosis. The dose used in these experiments approximates to those that will be used in future clinical studies and further supports the use of HP ^13^C‐fumarate as a probe for measuring tissue necrosis in a wide range of large animal models and in patients. However, we acknowledge the small sample size and variability of results between animals (Figure S5A–C) as a limitation of this work.

HP ^13^C‐fumarate has the potential to characterize cell death in many conditions such as cancer, ischemia, inflammation, transplant rejection, and infection, as well as to detect the necrosis associated with early response to a wide range of therapies in many diseases. Compared to existing preclinical HP ^13^C probes such as [1‐^13^C]pyruvate, the lack of a requirement for additional cofactors such as nicotinamide adenine dinucleotide  may be advantageous.^22^, ^27^ However, it is important to also consider that, despite the signal boost from having two labeled carbon atoms, the inequivalent C_1_ and C_4_ nuclei within [1,4‐^13^C_2_,2,3‐d_2_]malate have different chemical shifts, which restrict the maximum achievable SNR because the signals are not directly additive. The lower solubility of fumarate relative to pyruvate presents an additional limitation on the maximum achievable probe concentration at the point of injection, although the concentrations utilized here are higher than those employed for the recent successful clinical translation of HP ^13^C‐urea.^36^, ^51^ Recent preclinical studies have shown that higher concentrations of HP ^13^C‐fumarate may be achievable using parahydrogen‐induced polarization^53^ or using d‐DNP with a meglumine glassing matrix to improve solubility,^54^ although these approaches are yet to be scaled up to clinically relevant doses.

An important question for future research is the timing of the onset of necrosis because it is likely that HP ^13^C‐fumarate will be most sensitive shortly after the onset of membrane permeability but before established necrosis has set in, when FH may either be excreted from the extracellular space or undergo proteolysis. An analogy of this temporal change in metabolism can be found when HP ^13^C‐pyruvate is injected into patients undergoing neoadjuvant treatment for breast cancer patients: The subsequent ^13^C‐lactate signal produced may initially increase^55^ but then decreases after one cycle of treatment.^35^ Further information could be acquired by comparing the metabolism of dead cells using HP ^13^C‐fumarate with live cell imaging using HP ^13^C‐pyruvate or [^18^F]fluorodeoxyglucose uptake on positron emission tomography. Improvements in early detection of cellular necrosis in cancer and other diseases may have important implications for assessing experimental systemic treatments and monitoring existing therapies in the clinic.

[2,3‐d_2_]fumarate is an alternative probe that has been investigated with deuterium (^2^H) metabolic imaging (DMI) without the requirement for hyperpolarization.^43^, ^56^ DMI with oral ^2^H‐glucose has recently been translated to clinical field strength.^57^ Unlike HP ^13^C‐MRI, the ^2^H tissue signal does not decrease rapidly, facilitating its use for detecting slower metabolism,^56^ although the longer metabolite wash‐in time results in an increased time period for breakdown into further downstream products. In vivo linewidths are typically broader with DMI; therefore, SNR may be lower compared to ^13^C‐MRI. Preclinical studies using [2,3‐d_2_]fumarate have shown a decreased SNR but an enhanced malate/fumarate ratio when compared to HP [1,4‐^13^C_2_]fumarate.^43^, ^58^, ^59^ Future comparative studies of DMI and HP ^13^C DNP are required in a clinical setting to evaluate this in humans.

## 5 CONCLUSIONS

The work presented here demonstrates the evaluation and development of HP [1,4‐^13^C_2_,2,3‐d_2_]fumarate as a new clinical hyperpolarized probe. HP ^13^C‐fumarate has shown potential as an injectable MR contrast agent. We have undertaken the first‐in‐human injection of non‐HP ^13^C‐fumarate in healthy volunteers, optimized the hyperpolarization of the molecule and the imaging protocol used, and illustrated its applicability on clinical imaging systems in an in vivo large animal model of ischemia–reperfusion. This workflow mirrors the previous translational pathways for ^13^C‐labeled pyruvate,^30^, ^60^ urea,^36^, ^51^ and α‐ketoglutarate.^37^, ^61^ As with these emerging probes, the next stage in the translation of HP ^13^C‐fumarate is the imaging of HP ^13^C‐fumarate metabolism in healthy human volunteers and subsequently patients.

## CONFLICT OF INTEREST STATEMENT

The authors acknowledge research support from GE HealthCare. FAG has grants from AstraZeneca and NVision Imaging.

## Supporting information


**FIGURE S1.** Peripheral blood hematology and blood chemistry measurements obtained from the healthy human volunteer population before and after injection with ^13^C‐fumarate at various dose levels and flow rates.
**FIGURE S2.** Optimisation of microwave (A) frequency and (B) attenuation for hyperpolarization of ^13^C‐pyruvrate on the SPINlab hyperpolarizer. Vertical dashed red lines correspond to the values utilized in ^13^C‐fumarate experiments described in the main manuscript.
**FIGURE S3.** Polarization build‐up curves acquired from otherwise identical (0.38 g fumarate, 0.66 g DMSO formulation) samples of ^13^C‐fumarate as a function of AH111501 radical concentration utilized: (A) 17.5 mM; (B) 20 mM; (C) 25 mM; (D) 30 mM. Microwave frequency = 140.055 GHz, 8 dB attenuation, temperature = 0.8 K.
**FIGURE S4.** (A) Optical spectra acquired from a 1 mL sample of ^13^C‐fumarate as a function of time following addition of 5 UmL^−1^ FH; (B) corresponding ^13^C‐fumarate fraction of the total mixture at each time point as determined by area under curve integration of the optical spectra.
**FIGURE S5.** Spatial colormaps showing the measured malate‐to‐fumarate ratio in subjects #1 (A) and #5 (B); (C) Difference in mean malate‐to‐fumarate ratio between ischemic and healthy kidneys in all animals; (D) Histograms comparing malate‐to‐fumarate ratio in the ischemic and contralateral healthy kidneys for subject #6 (corresponding 2D MRSI colormap shown in Figure 7D of the main text).
**TABLE S1.** Cell cultures investigated in the ^13^C‐fumarate mutagenic potential study. NT, not tested.
**TABLE S2.** Statistical two‐tailed *t*‐test analysis of the effect of different ^13^C‐fumarate formulations on blood coagulation parameters (partial thromboplastin time, PT; activated partial thromboplastin time, APTT; Clauss Fibrinogen, FIBC). Values denoted with an asterisk represent statistically significant comparisons between blood‐to‐formulation ration of 1:2.70, which were not to be considered relevant for in vivo human imaging studies where the maximum infusion rate was defined to be 1:1.35. NVR, no valid result for this measurement.
**TABLE S3.** Sprague Dawley rat population groups investigated in the ^13^C‐fumarate toxicology study.
**TABLE S4.** ^13^C‐fumarate formulations investigated during the toxicology study.
**TABLE S5.** Statistically significant observations from peripheral blood hematology. Values listed as population means as recorded on Day 8 of the study (after receiving 4 total injections of either saline control, vehicle, or ^13^C‐fumarate). Asterisks denote *p*‐values <0.05 (*) or <0.01 (**) for comparisons against the saline control (Group 1). Hashes (# and ##) denote corresponding *p*‐values for comparison against the vehicle (Group 2). No statistically significant observations were observed between any groups for the following parameters: Hct, RBC, Retic, MCHC, MCV, RDW, Plt, PT, APTT. Additionally, no statistically significant observations were noted for any parameter from female rats in Group 3 (5 mg/kg 13C‐fumarate twice daily).
**TABLE S6.** Statistically significant observations from peripheral blood hematology during the second week of the recovery period. Values listed as population means. Asterisks denote *p*‐values <0.05 (*) or <0.01 (**) for comparisons against the saline control (Group 1). A hash (#) denotes corresponding *p*‐values for comparison against the vehicle (Group 2). No statistically significant observations were observed between any groups for the following parameters: Hct, RBC, Retic, MCH, WBC, N, L, E, B, M, LUC, PT, APTT (*t*‐test for Group 5 with Group 1 and Group 2, and for Group 2 with Group 1).
**TABLE S7.** Statistically significant observations from blood chemistry. Values listed as population means as recorded on Day 8 of the study (after receiving four total injections of either saline control, vehicle, or ^13^C‐fumarate). Asterisks denote *p*‐values <0.05 (*) or <0.01 (**) for comparisons against the saline control (Group 1). Hashes (# and ##) denote corresponding *p*‐values for comparison against the vehicle (Group 2). No statistically significant observations were observed between any groups for the following parameters: ALT, AST, Urea, Chol, Trig, Cl, Total Prot, A/G (Williams' test for Groups 3–5 with Group 1, *t*‐test for Group 2 with Group 1).
**TABLE S8.** Statistically significant observations from blood chemistry during the second week of the recovery period. Values listed as population means. Asterisks denote *p*‐values <0.05 (*) or <0.01 (**) for comparisons against the saline control (Group 1). No statistically significant comparisons against the vehicle control (Group 2) were observed. Additionally, no statistically significant observations were observed between any groups for the following parameters: ALT, AST, Create, Gluc, Trig, Na, K, Cl, Ca, Phos, Total Prot, Alb, A/G (*t*‐test for Group 5 with Group 1 and Group 2, and for Group 2 with Group 1).
**TABLE S9.** Statistically significant observations from urinalysis during the second week of the recovery period. Values listed as population means. Asterisks denote *p*‐values <0.05 (*) or <0.01 (**) for comparisons against the saline control (Group 1). No statistically significant comparisons against the vehicle control (Group 2) were observed. Additionally, no corresponding statistically significant observations were observed between any groups for any parameters in the male populations.
**TABLE S10.** Statistically significant observations from organ weighing at necropsy. Values listed as adjusted population means as recorded on Day 8 of the study (after receiving four total injections of either saline control, vehicle, or ^13^C‐fumarate). Asterisks denote *p*‐values <0.05 (*) or <0.01 (**) for comparisons against the saline control (Group 1). A hash (#) denotes corresponding *p*‐values for comparison against the vehicle (Group 2). No statistically significant observations were observed between any groups for terminal body weight, nor for the following organs: adrenals, brain, heart, kidneys, ovaries, pituitary, seminal vesicles, spleen, testes, thymus, thyroid or uterus. Additionally, no statistically significant observations were noted for any organ within the male rat populations in Group 3 (5 mg/kg ^13^C‐fumarate twice daily) and Group 4 (10 mg/kg ^13^C‐fumarate twice daily).
**TABLE S11.** Statistically significant observations from organ weighing at necropsy. Values listed as adjusted population means as recorded during the second week of the recovery period. Asterisks denote *p*‐values <0.05 (*) or <0.01 (**) for comparisons against the saline control (Group 1). A hash denotes corresponding *p*‐values for comparison against the vehicle (Group 2). No statistically significant observations were observed between any groups for the following organs: adrenals, brain, epididymides, heart, kidneys, ovaries, pituitary, prostate, seminal vesicles, spleen or uterus.
**TABLE S12.** Repeatability of ^13^C‐fumarate formulation to various target dose concentrations in the healthy human volunteer injections. 0.96 mg/kg corresponds to a concentration of 20 mM, 1.92 mg/kg to 40 mM, and 3.84 mg/kg to 80 mM.
