# Supplementary material for: A pathway toward clinical translation of hyperpolarized [1,4‐ 13C2 ,2,3‐d2]fumarate as an imaging biomarker for early cellular necrosis in vivo
Source: Magn Reson Med. 2025 May 7;94(3):1202–17. doi: 10.1002/mrm.30519 (PMC12202726; doi:10.1002/mrm.30519)
Supplement: Supplementary file 1 — FIGURE S1. Peripheral blood hematology and blood chemistry measurements obtained from the healthy human volunteer population before and after injection with 13C‐fumarate at various dose levels and flow rates. FIGURE S2. Optimisation of microwave (A) frequency and (B) attenuation for hyperpolarization of 13C‐pyruvrate on the SPINlab hyperpolarizer. Vertical dashed red lines correspond to the values utilized in 13C‐fumarate experiments described in the main manuscript. FIGURE S3. Polarization build‐up curves acquired from otherwise identical (0.38 g fumarate, 0.66 g DMSO formulation) samples of 13C‐fumarate as a function of AH111501 radical concentration utilized: (A) 17.5 mM; (B) 20 mM; (C) 25 mM; (D) 30 mM. Microwave frequency = 140.055 GHz, 8 dB attenuation, temperature = 0.8 K. FIGURE S4. (A) Optical spectra acquired from a 1 mL sample of 13C‐fumarate as a function of time following addition of 5 UmL−1 FH; (B) corresponding 13C‐fumarate fraction of the total mixture at each time point as determined by area under curve integration of the optical spectra. FIGURE S5. Spatial colormaps showing the measured malate‐to‐fumarate ratio in subjects #1 (A) and #5 (B); (C) Difference in mean malate‐to‐fumarate ratio between ischemic and healthy kidneys in all animals; (D) Histograms comparing malate‐to‐fumarate ratio in the ischemic and contralateral healthy kidneys for subject #6 (corresponding 2D MRSI colormap shown in Figure 7D of the main text). TABLE S1. Cell cultures investigated in the 13C‐fumarate mutagenic potential study. NT, not tested. TABLE S2. Statistical two‐tailed t‐test analysis of the effect of different 13C‐fumarate formulations on blood coagulation parameters (partial thromboplastin time, PT; activated partial thromboplastin time, APTT; Clauss Fibrinogen, FIBC). Values denoted with an asterisk represent statistically significant comparisons between blood‐to‐formulation ration of 1:2.70, which were not to be considered relevant for in vivo human imaging stu [file MRM-94-1202-s001.docx]

SUPPORTING INFORMATION

A pathway towards clinical translation of hyperpolarized [1,4-^13^C_2_,2,3-d_2_]fumarate as an imaging biomarker for early cellular necrosis in vivo

Jonathan R. Birchall*^1^, Pascal Wodtke*^1,2^, Ashley Grimmer^1,2^, Esben S. S. Hansen^3^, Lotte B. Bertelsen^3^, Nikolaj Bøgh^3^, Marta Wylot^1^, Maria J. Zamora-Morales^1^, Otso Arponen^1,4^, Ines Horvat-Menih^1^, Elizabeth C. Latimer^1^, Fung Tan^5^, Evita Pappa^6^, Johann Graggaber^7^, Joseph Cheriyan^6,7^, Kelly Holmes^1^, Matthew J. Locke^1^, Helen Sladen^2^, Joan Boren^1,8^, Mikko I. Kettunen^2,9^, Anita Chhabra^5^, Ian B. Wilkinson^6,7^, Christoffer Laustsen^3^, Kevin Brindle^2^, Mary A. McLean** ^1,2^ and Ferdia A. Gallagher**^1,2^

^1^ University of Cambridge Department of Radiology, Cambridge CB2 0QQ, United Kingdom

^2^ Cancer Research UK Cambridge Institute, Cambridge CB2 0RE, United Kingdom

^3^ Aarhus University MR Research Centre, Department of Clinical Medicine, 8200 Aarhus N, Denmark

^4^ Institute of Clinical Medicine, University of Eastern Finland, 70210 Kuopio, Finland

^5^ Radiopharmacy Department, Cambridge University Hospitals NHS Foundation Trust, Cambridge CB2 0QQ, United Kingdom

^6^ University of Cambridge Department of Medicine, Division of Experimental Medicine and Immunotherapeutics, Cambridge CB2 0QQ, United Kingdom

^7^ Cambridge Clinical Trials Unit, Cambridge University Hospitals NHS Trust, Cambridge CB2 0SL, United Kingdom

^8^ The Discovery Centre, AstraZeneca, Cambridge Biomedical Campus, Cambridge CB2 0AA, United Kingdom

^9^ A.I. Virtanen Institute for Molecular Sciences, University of Eastern Finland, 70210 Kuopio, Finland

* JB and PW contributed equally to this work

** MM and FG contributed equally to this work

**Table of Contents**

[1) Mutagenic activity study methodology 3](#_Toc189644134)

[2) Statistically significant hemocompatability observations 4](#_Toc189644135)

[3) Animal toxicology study methodology 6](#_Toc189644136)

[4) Statistically significant toxicity observations 8](#_Toc189644137)

[5) Validation of ^13^C-fumarate concentration and tolerability in healthy human volunteers 14](#_Toc189644138)

[6) [1-^13^C]pyruvate microwave frequency and amplitude sweep 16](#_Toc189644139)

[7) Polarization build-up as a function of radical concentration 17](#_Toc189644140)

[8) Estimation of fumarate polarization in phantom studies 18](#_Toc189644141)

[9) Optical spectroscopic characterization of fumarate hydratase enzyme activity 19](#_Toc189644142)

[10) MRSI characterization of malate-to-fumarate ratio in vivo 20](#_Toc189644143)

[11) References used in Supporting Information 22](#_Toc189644144)

# Mutagenic activity study methodology

The mutagenic potential of exogenous ^13^C-fumarate was assessed in L5178Y mouse lymphoma cells (ATCC, Manassas VA) incubated in both the absence and presence of supplemented rat liver homogenate fraction (S9 mix) to simulate isolated and enzyme-metabolized systems. ^13^C-fumarate was formulated in DMSO at concentrations from 0.23 to 120.03 μg/mL, and cells were incubated at 34 to 39 °C for either 3 or 24 hours in the absence of S9 mix, or 3 hours in the presence of S9 mix. Cultures contained ~ 1 x 10^7^ cells in a final volume of 10 mL. Cultures containing methyl methanesulphonate (MMS) and benzo[a]pyrene (B[a]P) served as positive controls for mutagenic activity.

Following the treatment period, cells were sampled after 24 and 48 hours to assess for precipitation. After 48 hours, cultures with a density > 1 x 10^5^ cells per mL were assessed by plating 1.6 cells per well in a humidified incubator at 34 to 39 °C in a 5% CO_2_ in air atmosphere for 10 to 12 days. The number of empty wells was recorded, and the cloning efficiency (CE) for each culture was calculated by comparing the fraction of empty wells relative to a mean control culture with no ^13^C-fumarate present.

The relative total growth (RTG) was calculated by taking the ratio of post-treatment cell counts for each culture relative to a mean control culture with no ^13^C-fumarate present, and then multiplying by the ratio of post-treatment CE relative to the mean control culture. No substantial reduction in RTG was observed in any formulation and at any ^13^C-fumarate concentration. There were no increases in the mean mutant frequencies (MF, per 10^6^ surviving cells) of any of the test concentrations assessed that exceeded the sum of the mean concurrent vehicle control MF and the Global Evaluation Factor (GEF, 126 x 10^-6^ for microwell assays), within acceptable levels of toxicity. Conversely, increases in mutation frequency beyond these limits were observed in the positive control cultures. A summary of these results is presented in Table S1.

| Test Item | Dose Level  μg/mL | 3-hour Treatment  (no S9 mix) | | 3-hour Treatment  (with S9 mix) | | 24-hour Treatment  (no S9 mix) | |
| --- | --- | --- | --- | --- | --- | --- | --- |
|  |  | Mean RTG  (%) | Mean MF  (x10^-6^) | Mean RTG  (%) | Mean MF  (x10^-6^) | Mean RTG  (%) | Mean MF  (x10^-6^) |
| Control (DMSO) | 0 | 100 | 60 | 100 | 70 | 100 | 52 |
| ^13^C-fumarate | 7.5 | 81 | 74 | 72 | 72 | 94 | 53 |
|  | 15 | 94 | 83 | 83 | 74 | 86 | 62 |
|  | 30 | 91 | 81 | 78 | 63 | 93 | 61 |
|  | 60 | 85 | 65 | 83 | 73 | 97 | 51 |
|  | 120 | 83 | 54 | 101 | 58 | 81 | 59 |
| MMS | 10 | 50 | 521 | NT | NT | NT | NT |
|  | 5 | NT | NT | NT | NT | 45 | 755 |
| B[a]P | 1.5 | NT | NT | 40 | 870 | NT | NT |

Table S1: Cell cultures investigated in the ^13^C-fumarate mutagenic potential study. NT = not tested.

# Statistically significant hemocompatability observations

Whilst there was no evidence for mutagenic potential of ^13^C-fumarate noted, some statistically significant deviations between ^13^C-fumarate-administered and control population groups were observed in the *in vitro* hemocompatability study at the planned maximum in vivo infusion rate of 1 mL blood to 1.35 mL of ^13^C-fumarate formulation. These changes—described in the main text—were considered not to be clinically significant but are included here in Table S2 for completeness. Tube IDs with even numbers were formulated with double the planned maximum in vivo blood-to-formulation ratio as a safety test.

| Treatment | Pool ID | Tube ID | PT (Sec) | APTT (Sec) | FibC (gL^-1^) |
| --- | --- | --- | --- | --- | --- |
| Fum. 12.0 mgmL^-1^  Ratio 1 : 1.35 | 1 | 01 | 27.1 | 20.8 | 0.81 |
|  | 2 |  | 28.5 | 23.5 | 0.67 |
|  | 3 |  | 30.1 | 23.9 | 0.78 |
|  | Mean |  | 28.6 | 22.7 | 0.75 |
| Fum. 12.0 mgmL^-1^  Ratio 1 : 2.70 | 1 | 02 | 42.3 | 34.5 | 2.95 |
|  | 2 |  | 44.9 | 34.4 | NVR |
|  | 3 |  | 46.2 | 29.3 | NVR |
|  | Mean |  | 44.5 * | 32.7 * | 2.95 |
| Negative Control  Ratio 1 : 1.35 | 1 | 03 | 27.5 | 19.7 | 0.90 |
|  | 2 |  | 27.6 | 21.2 | 0.99 |
|  | 3 |  | 27.8 | 21.3 | 0.76 |
|  | Mean |  | 27.6 | 20.7 | 0.88 |
| Negative Control  Ratio 1 : 2.70 | 1 | 04 | 39.2 | 23.7 | 2.84 |
|  | 2 |  | 39.9 | 21.5 | NVR |
|  | 3 |  | 41.3 | 25.7 | NVR |
|  | Mean |  | 40.1 * | 23.6 * | 2.84 |
| Vehicle  Ratio 1 : 1.35 | 1 | 05 | 22.0 | 20.8 | 0.93 |
|  | 2 |  | 23.5 | 22.9 | 0.77 |
|  | 3 |  | 24.7 | 23.4 | 0.74 |
|  | Mean |  | 23.4 ^a,b,c^ | 22.4 ^c^ | 0.81 |
| Vehicle  Ratio 1 : 2.70 | 1 | 06 | 33.9 | 28.7 | NVR |
|  | 2 |  | 36.4 | 30.3 | 5.80 |
|  | 3 |  | 38.5 | 30.8 | NVR |
|  | Mean |  | 36.3 * | 29.9 | 5.80 |

Table S2: Statistical two-tailed t-test analysis of the effect of different ^13^C-fumarate formulations on blood coagulation parameters (partial thromboplastin time, PT; activated partial thromboplastin time, APTT; Clauss Fibrinogen, FIBC). Values denoted with an asterisk represent statistically significant comparisons between blood-to-formulation ration of 1:2.70, which were not to be considered relevant for ­in vivo human imaging studies where the maximum infusion rate was defined to be 1:1.35. NVR = no valid result for this measurement.

As discussed in the main text, it is suggested that ^13^C-fumarate and vehicle (1.8% DMSO, 0.08% AH111501, 16.1% Trometamol buffer in water for injections) at a blood-to-formulation ratio of 1:1.35 may impact coagulation parameters (APTT, PT and FibC). This is evidenced by observations a-c listed in Table S2 above:

1. Tube 5 (Vehicle) vs Tube 1 (Formulation) — p < 0.01
2. Tube 5 (Vehicle) vs Tube 3 (Negative Control) — p < 0.01
3. Tube 5 (Vehicle) vs Tube 6 (Vehicle @ 2x infusion rate) — p < 0.001

However, platelet counts measured in tubes containing ^13^C-fumarate and vehicle at a blood-to-formulation ratio of 1:1.35 were comparable to the negative control, and a visual comparison of the blood film showed no platelet clumping or other evidence of coagulation. In addition, there was no evidence of hemolysis, suggesting that whilst the vehicle alone increased PT, statistically there is no biological significance as the ^13^C-fumarate formulations had little effect on hemocompatibility.

# Animal toxicology study methodology

Figure 3 of the main text illustrates the effect of exogenous ^13^C-fumarate administration on mean ^13^C-fumarate blood plasma concentration at different dose levels in the animal population investigated. Formulations comprised a specified ^13^C-fumarate dose and an injection vehicle as follows: 1.8% DMSO, 0.08% AH111501, 16.1% trometamol buffer in water. The radical concentration was 20 mM for all injections.

To ensure that the observations reported in the toxicology study were linked to administration of ^13^C-fumarate and not the injection vehicle, two control groups were also assessed, containing either only the vehicle, or a saline solution of the same total volume. A summary of the animal groups investigated in this study is shown below in Table S3. All animals were aged approximately 8-9 weeks at the start of treatment. Male weights ranged from 223 to 413 g, whilst female weights ranged from 179 to 278 g.

Blood samples acquired from three male and three female subjects in each of the ^13^C-fumarate treatment groups (Groups 3-5) were used to inform on the dynamic change in toxicokinetic parameters following repeated injections, as outlined in Methods Section 2 of the main manuscript (see Figure 3 for results).

| Group | Treatment | Fumarate dose (mgkg^-1^ / day) | Number of animals | | | | | |
| --- | --- | --- | --- | --- | --- | --- | --- | --- |
|  |  |  | Main study | | Recovery phase | | Toxicokinetic study | |
|  |  |  | Male | Female | Male | Female | Male | Female |
| 1 | Saline control | 0 | 10 | 10 | 5 | 5 | 3 | 3 |
| 2 | Vehicle control | 0 | 10 | 10 | 5 | 5 | 3 | 3 |
| 3 | ^13^C-fumarate | 10 | 10 | 10 | - | - | 6 | 6 |
| 4 | ^13^C-fumarate | 60 | 10 | 10 | - | - | 6 | 6 |
| 5 | ^13^C-fumarate | 120 | 10 | 10 | 5 | 5 | 6 | 6 |

Table S3: Sprague Dawley rat population groups investigated in the ^13^C-fumarate toxicology study.

No adverse clinical signs were observed in either of the control groups besides green urine (which was also observed in all groups which received a ^13^C-fumarate injection). This observation was linked to the green color of the AH111501 excipient used in the vehicle and was of no toxicological significance: in human studies, this radical was removed by filtration prior to injection.

Formulations investigated in the toxicology study were created by initially dissolving the required amount of ^13^C-fumarate in the appropriate quantity of DMSO. Remaining vehicle excipients were gradually added once dissolved, followed by water, and either NaOH or HCl as required to ensure a suitable pH (6.0 – 8.2) for injection at a volume of 5 mLkg^-1^. A summary of the various dose levels used, and their formulation, is shown below in Table S4.

| Group | Treatment | Fumarate dose (mgkg^-1^ / day) | Nominal conc. (mgmL^-1^) | Formulated conc. (mgmL^-1^) | Volume injected (mLkg^-1^ / dose) |
| --- | --- | --- | --- | --- | --- |
| 1 | Saline control | 0 | 0 | 0 | 5 |
| 2 | Vehicle control | 0 | 0 | 0 | 5 |
| 3 | ^13^C-fumarate | 10 | 1 | 1 | 5 |
| 4 | ^13^C-fumarate | 60 | 6 | 6 | 5 |
| 5 | ^13^C-fumarate | 120 | 12 | 12 | 5 |

Table S4: ^13^C-fumarate formulations investigated during the toxicology study.

The molecular weight of endogenous fumarate is 116 gmol^-1^, which rises to 120 gmol^-1^ for the isotopologue with two ^13^C and ^2^H nuclear sites used in this work. The maximum concentration *C* of ^13^C-fumarate validated for injection (in the 12 mgmL^-1^ dose level group) was 100 mM as determined from Equation S1:

$C=\frac{n}{V}=\frac{m}{MV}= \frac{120}{120*10}=0.1 M$ (S1)

Where *n* is the molar quantity (mass *m* divided by molar mass *M*) of ^13^C-fumarate injected per kg, and V is the total dose volume per kg (5 mLkg^-1^ twice daily = 10 mLkg^-1^ total dose). It should be noted that despite the much larger maximum permissible injection volume (5 mLkg^-1^) compared to that utilised in the human studies (0.4 – 0.6 mLkg^-1^), the maximum achievable concentration formulated for injection is the same.

# Statistically significant toxicity observations

A limited number of statistically significant observations were noted from the animal toxicity study of ^13^C-fumarate, mostly at the highest dose models of 120 mgkg^-1^ per day. These findings were considered not clinically relevant and/or were observed to be reversible after the allocated two-week recovery period but are included here for completeness. These findings are categorized as follows: peripheral blood hematology (Tables S5 and S6), blood chemistry (Tables S7 and S8), urinalysis (Table S9) and organ weights (Tables S10 and S11). For group classification pertaining to the injection (saline control, vehicle, or ^13^C-fumarate) administered in each case, please refer to Table S3. All inferences for statistical significance were derived from Williams’ test (for Groups 3-5 with Group 1) and t-test (for Group 2 with Group 1) unless specified otherwise. The population sizes in each case are N = 20 (10M/10F) for observations recorded on day 8 after the final injection, and N = 10 (5M/5F) for observations recorded following two weeks of recovery.

- 1. Peripheral blood hematology

Parameters assessed are listed as follows: hematocrit (Hct), hemoglobin concentration (Hb), erythrocyte count (RBC), absolute reticulocyte count (Retic), mean cell hemoglobin (MCH), mean cell hemoglobin concentration (MCHC), mean cell volume (MCV), red cell distribution width (RDW), total leucocyte count (WBC), platelet count (Plt), prothrombin time (PT) and activated partial thromboplastin time (APTT). Leucocytes were further differentiated as: neutrophils (N), lymphocytes (L), eosinophils (E), basophils (B), monocytes (M), large unstained cells (LUC). Statistically significant observations from measurements taken after the second injection on day 8 of the study are listed below in Table S5:

| Group  No./Sex | Hb  g/dL | MCH  pg | WBC  x10^9^/L | N  x10^9^/L | L  x10^9^/L | E  x10^9^/L | B  x10^9^/L | M  x10^9^/L | LUC  x10^9^/L |
| --- | --- | --- | --- | --- | --- | --- | --- | --- | --- |
| 1/M | 14.3 | 19.6 | 8.91 | 0.97 | 7.48 | 0.07 | 0.05 | 0.25 | 0.10 |
| 2/M | 14.5 | 19.8 | 9.04 | 1.23 | 7.29 | 0.11 | 0.03 | 0.27 | 0.12 |
| 3/M | 14.5 | 19.7 | 11.60*# | 1.32 | 9.73*# | 0.09 | 0.07 | 0.28 | 0.13 |
| 4/M | 14.5 | 19.7 | 10.50*# | 1.01 | 9.03*# | 0.08 | 0.04 | 0.22 | 0.12 |
| 5/M | 14.8 | 20.1 | 12.67**## | 1.34 | 10.67**## | 0.11* | 0.07## | 0.31 | 0.17**# |
| 1/F | 14.1 | 19.9 | 6.38 | 0.67 | 5.39 | 0.08 | 0.03 | 0.12 | 0.08 |
| 2/F | 14.2 | 20.0 | 8.77* | 1.03** | 7.30* | 0.10 | 0.04 | 0.21** | 0.09 |
| 4/F | 13.8 | 19.2# | 9.75** | 0.93 | 8.32** | 0.12 | 0.04 | 0.22** | 0.11 |
| 5/F | 14.0 | 19.6# | 10.95**# | 1.13** | 9.30**# | 0.12 | 0.05** | 0.24** | 0.13**# |

**Table S5:** Statistically significant observations from peripheral blood hematology. Values listed as population means as recorded on Day 8 of the study (after receiving 4 total injections of either saline control, vehicle, or ^13^C-fumarate). Asterisks denote p-values < 0.05 (*) or < 0.01 (**) for comparisons against the saline control (Group 1). Hashes (# and ##) denote corresponding p-values for comparison against the vehicle (Group 2). No statistically significant observations were observed between any groups for the following parameters: Hct, RBC, Retic, MCHC, MCV, RDW, Plt, PT, APTT. Additionally, no statistically significant observations were noted for any parameter from female rats in Group 3 (5 mg/kg ^13^C-fumarate twice daily).

At the end of the treatment period, higher group mean lymphocyte, monocyte and large unstained cell counts, and consequently total white blood cell count were observed in males receiving 120 mg/kg per day and females receiving 60 or 120 mg/kg per week in comparison with the saline or vehicle controls. The extent of the difference from controls for each parameter was minor but consistent for most animals in these groups. There was also an increase in mean lymphocyte count in males receiving 10 or 60 mg/kg/day, but there was no dose response relationship. The observations were not associated with any histopathological evidence of an inflammatory response and were thus considered not to be adverse.

In addition to the hematological work performed on Day 8, further blood samples were collected following two weeks of recovery from the control populations (Group 1 and 2) as well as the highest ^13^C-fumarate dose population (Group 5, 60 mg/kg ^13^C-fumarate twice daily). Statistically significant observations from these measurements are listed below in Table S6 (all t-test except for MCHC which was assessed using Wilcoxon rank sum test):

| Group  No./Sex | Hb  g/dL | MCHC  pg | MCV  fL | RDW  % | Plt  x10^9^/L |
| --- | --- | --- | --- | --- | --- |
| 1/M | 14.7 | 34.3 | 58.4 | 11.7 | 897 |
| 2/M | 15.2 | 33.7 | 60.5* | 10.8** | 904 |
| 5/M | 15.2 | 33.7* | 59.7 | 10.8** | 819 |
| 1/F | 14.3 | 20.1 | 57.3 | 10.4 | 1076 |
| 2/F | 14.3 | 20.2 | 57.8 | 10.1 | 1036 |
| 5/F | 14.7# | 20.1 | 57.3 | 10.2 | 928* |

**Table S6:** Statistically significant observations from peripheral blood hematology during the second week of the recovery period. Values listed as population means. Asterisks denote p-values < 0.05 (*) or < 0.01 (**) for comparisons against the saline control (Group 1). A hash (#) denotes corresponding p-values for comparison against the vehicle (Group 2). No statistically significant observations were observed between any groups for the following parameters: Hct, RBC, Retic, MCH, WBC, N, L, E, B, M, LUC, PT, APTT (t-test for Group 5 with Group 1 and Group 2, and for Group 2 with Group 1).

By the end of the two-week recovery period, the effect on white blood cell count was no longer evident, showing full recovery. There were no other effects on hematology or coagulation parameters.

- 1. Blood biochemistry

Parameters assessed are listed as follows: alkaline phosphatase (ALP), alanine aminotransferase (ALT), aspartate aminotransferase (AST), total bilirubin (Bili), urea, creatinine (Creat), glucose (Gluc), total cholesterol (Chol), triglycerides (Trig), sodium (Na), potassium (K), chloride (Cl), calcium (Ca), inorganic phosphorus (Phos), total protein (Total Prot), albumin (Alb) and albumin/globulin ratio (A/G). Statistically significant observations from measurements taken after the second injection on day 8 of the study are listed in Table S7 below. Bilirubin concentration correlations between ^13^C-fumarate groups and Groups 1 and 2 were assessed using Fisher’s exact test. Calcium concentration correlations in males and potassium concentration correlations in females were assessed using Shirley’s test for ^13^C-fumarate groups vs. Group 1, and Wilcoxon rank sum test to compare Groups 1 and 2. Calcium concentration correlations in females was assessed using Dunnet’s test (^13^C-fumarate groups vs. Groups 1 and 2) and a t-test for Group 1 with Group 2.

| Group  No./Sex | ALP  U/L | Bili  μmol/L | Creat  μmol/L | Gluc  mmol/L | Na  mmol/L | K  mmol/L | Ca  mmol/L | Phos  mmol/L | Alb  g/L |
| --- | --- | --- | --- | --- | --- | --- | --- | --- | --- |
| 1/M | 140 | 1 | 25 | 9.41 | 141 | 5.16 | 2.54 | 1.97 | 36 |
| 2/M | 153 | 1 | 26 | 9.26 | 141 | 5.10 | 2.50 | 1.86 | 36 |
| 3/M | 144 | 1 | 22# | 10.12 | 141 | 5.07 | 2.52 | 1.89 | 36 |
| 4/M | 181* | 1 | 24# | 10.07 | 142 | 4.98 | 2.63# | 2.09## | 37 |
| 5/M | 167* | 1 | 24# | 9.86 | 142 | 4.82 | 2.64*## | 2.14*## | 36 |
| 1/F | 75 | 1 | 31 | 8.28 | 139 | 4.92 | 2.57 | 1.57 | 41 |
| 2/F | 81 | 2 | 29 | 8.92 | 139 | 4.53 | 2.56 | 1.60 | 41 |
| 3/F | 96 | 1 | 31 | 8.65 | 141* | 4.42 | 2.47**## | 1.63 | 38*# |
| 4/F | 88 | 2** | 31 | 9.39* | 140* | 4.45 | 2.53 | 1.57 | 39*# |
| 5/F | 107**## | 2**# | 31 | 8.85* | 141* | 4.11**# | 2.57 | 1.80**## | 40*# |

**Table S7:** Statistically significant observations from blood chemistry. Values listed as population means as recorded on Day 8 of the study (after receiving 4 total injections of either saline control, vehicle, or ^13^C-fumarate). Asterisks denote p-values < 0.05 (*) or < 0.01 (**) for comparisons against the saline control (Group 1). Hashes (# and ##) denote corresponding p-values for comparison against the vehicle (Group 2). No statistically significant observations were observed between any groups for the following parameters: ALT, AST, Urea, Chol, Trig, Cl, Total Prot, A/G (Williams’ test for Groups 3-5 with Group 1, t-test for Group 2 with Group 1).

At the end of the treatment period, higher group mean alkaline phosphatase activity was observed in males receiving 60 or 120 mg/kg ^13^C-fumarate per week (approximately 29% and 19%, respectively), and in the females receiving 120 mg/kg ^13^C-fumarate per day (approximately 43%) compared to the saline controls. There was no clear dose relationship and individual values showed a high degree of variation. There were also some variations in mean plasma electrolyte concentrations compared to controls: high calcium and phosphorus concentrations in males receiving 60 or 120 mg/kg ^13^C-fumarate per day, and high phosphorus and low potassium concentrations in females receiving 120 mg/kg ^13^C-fumarate per day.

To correlate with the peripheral blood hematology, additional blood chemistry observations were recorded during the second week of recovery in Groups 1, 2 and 5. Statistically significant observations from these measurements are listed below in Table S8, all obtained using a t-test:

| Group  No./Sex | ALP  U/L | Bili  μmol/L | Urea  mmol/L | Chol  mmol/L |
| --- | --- | --- | --- | --- |
| 1/M | 176 | 2 | 4.93 | 1.43 |
| 2/M | 173 | 1* | 4.42* | 1.80 |
| 5/M | 168 | 1* | 4.34* | 1.63 |
| 1/F | 81 | 1 | 5.54 | 2.12 |
| 2/F | 112* | 1 | 5.96 | 1.57* |
| 5/F | 95 | 1 | 5.24 | 1.80 |

**Table S8:** Statistically significant observations from blood chemistry during the second week of the recovery period. Values listed as population means. Asterisks denote p-values < 0.05 (*) or < 0.01 (**) for comparisons against the saline control (Group 1). No statistically significant comparisons against the vehicle control (Group 2) were observed. Additionally, no statistically significant observations were observed between any groups for the following parameters: ALT, AST, Create, Gluc, Trig, Na, K, Cl, Ca, Phos, Total Prot, Alb, A/G (t-test for Group 5 with Group 1 and Group 2, and for Group 2 with Group 1).

By the end of the two-week recovery period, the above differences from the control values were no longer evident. All other inter-group differences from controls were minimal, lacked dose-relationship or were confined to one sex and were considered to reflect normal biological variation.

- 1. Urinalysis

A potentially statistically significant change (p < 0.01) in urine specific gravity (SG) was observed after the second injection on day 8 of the study for male populations receiving the vehicle (Group 2, 1037±4 g/L) and the highest ^13^C-fumarate dose (Group 5, 1037±5 g/L) relative to the male control population (Group 1, 1032±3 g/L), as assessed using Wilcoxon rank sum test, but no statistical significance was observed between the vehicle control and ^13^C-fumarate dose population groups. No statistically significant change in specific gravity were observed for the corresponding female populations, and neither were any statistically significant changes in volume (Vol) or pH observed.

To correlate with the other measurements, additional urinalysis was performed during the second week of recovery in Groups 1, 2 and 5. Statistically significant observations from these measurements are listed below in Table S9, all obtained using a t-test:

| Group  No. /Sex | Vol  mL | pH | S/G  g/L |
| --- | --- | --- | --- |
| 1/F | 3.1 | 6.1 | 1049 |
| 2/F | 5.2** | 6.4* | 1032* |
| 5/F | 5.6** | 6.3 | 1034* |

**Table S9:** Statistically significant observations from urinalysis during the second week of the recovery period. Values listed as population means. Asterisks denote p-values < 0.05 (*) or < 0.01 (**) for comparisons against the saline control (Group 1). No statistically significant comparisons against the vehicle control (Group 2) were observed. Additionally, no corresponding statistically significant observations were observed between any groups for any parameters in the male populations.

All males receiving 120 mg/kg per week and 8 of 10 males receiving 10 mg/kg per week had higher ketone levels in urine samples collected at the end of the treatment period in comparison with 4 of 10 seen in the control groups. There was no effect on urine composition for males dosed at 60 mg/kg per day, or in the female populations. By the end of the two-week recovery period, ketone levels in the urine of males previously dosed at 120 mg/kg per day was similar to the saline control values.

- 1. Organ Weights

Organs were removed and weighed at necropsy following asphyxiation with carbon dioxide and subsequent exsanguination. In addition to the terminal total body weight, organs weighed are listed as follows: adrenals, brain, epididymides, heart, kidneys, liver, ovaries, pituitary, prostate, seminal vesicles, spleen, testes, thymus, thyroid and uterus. Statistically significant organ weight observations from animals killed following the second injection on day 8 of the study are listed in Table S10 below.

| Group  No./Sex | Epididymides  g | Liver  g | Thymus  g | Prostate  g |
| --- | --- | --- | --- | --- |
| 1/M | 0.951 | 12.319 | 0.476 | 0.751 |
| 2/M | 0.978 | 12.611 | 0.421 | 0.777 |
| 5/M | 1.046* | 12.658 | 0.518 | 0.951*# |
| 1/F | N/A | 7.818 | 0.410 | N/A |
| 2/F | N/A | 8.355 | 0.435 | N/A |
| 3/F | N/A | 8.572* | 0.450 | N/A |
| 4/F | N/A | 8.864** | 0.439 | N/A |
| 5/F | N/A | 8.870** | 0.470 | N/A |

**Table S10:** Statistically significant observations from organ weighing at necropsy. Values listed as adjusted population means as recorded on Day 8 of the study (after receiving four total injections of either saline control, vehicle, or ^13^C-fumarate). Asterisks denote p-values < 0.05 (*) or < 0.01 (**) for comparisons against the saline control (Group 1). A hash (#) denotes the corresponding p-values for comparison against the vehicle (Group 2). No statistically significant observations were observed between any groups for terminal body weight, nor for the following organs: adrenals, brain, heart, kidneys, ovaries, pituitary, seminal vesicles, spleen, testes, thymus, thyroid or uterus. Additionally, no statistically significant observations were noted for any organ within the male rat populations in Group 3 (5 mg/kg ^13^C-fumarate twice daily) and Group 4 (10 mg/kg ^13^C-fumarate twice daily).

At the end of the treatment period, group mean absolute and body weight-adjusted thymus weights were slightly higher than controls for both sexes in the highest ^13^C-fumarate dose group, with adjusted values approximately 9% and 15% higher for the males and females, respectively compared with the saline controls. Group mean absolute and body weight-adjusted prostate weights were higher than saline controls for the males given 60 or 120 mg/kg per day, with adjusted values 13% and 27% higher, respectively. Group mean absolute and body weight-adjusted liver weights were slightly higher than saline or vehicle controls for females given 60 or 120 mg/kg per day, with adjusted values approximately 13% higher than saline control values. Higher prostate weights observed in males given 60 or 120 mg/kg/day similarly did not correlate with any histopathological change in this organ or other organs in the male reproductive tract, were not apparent at the end of the recovery period and were therefore considered not to be adverse. Corresponding measurements of organ weight following two weeks of recovery exhibiting statistical significance (all determined using t-test) are shown below in Table S11:

| Group  No./Sex | Terminal body weight  g | Liver  g | Testes  g | Thymus  g | Thyroid  g |
| --- | --- | --- | --- | --- | --- |
| 1/M | 339.8 | 13.514 | 3.283 | 0.503 | 0.019 |
| 2/M | 336.7 | 12.102* | 3.465 | 0.524 | 0.015 |
| 5/M | 319.8 | 11.040** | 3.142# | 0.412# | 0.018 |
| 1/F | 219.9 | 8.798 | N/A | 0.433 | 0.017 |
| 2/F | 222.2 | 8.327 | N/A | 0.477 | 0.013 |
| 5/F | 232.8* | 8.629 | N/A | 0.468 | 0.011* |

**Table S11:** Statistically significant observations from organ weighing at necropsy. Values listed as adjusted population means as recorded during the second week of the recovery period. Asterisks denote p-values < 0.05 (*) or < 0.01 (**) for comparisons against the saline control (Group 1). A hash (#) denotes corresponding p-values for comparison against the vehicle (Group 2). No statistically significant observations were observed between any groups for the following organs: adrenals, brain, epididymides, heart, kidneys, ovaries, pituitary, prostate, seminal vesicles, spleen or uterus.

The slightly higher liver weights seen in female rats that received 60 or 120 mg/kg injections of ^13^C-fumarate correlated with slightly higher plasma alkaline phosphatase (ALP) activity at the highest dose level. Higher liver weight in the absence of a histological change which is then shown to revert following a treatment free period is believed to be linked to adaptive responses involving enzyme induction (potentially involving ALP) to facilitate clearance of ^13^C-fumarate. This finding was regarded as non-adverse.

By the end of the two-week recovery period the remaining differences from the control values were no longer evident, and generally similar to the saline control values. All other inter-group differences from controls were minimal, lacked dose-relationship and were considered to reflect normal biological variation rather than an effect of ^13^C-fumarate administration.

- 1. Macropathology

In addition to organ weighing, a macroscopic visual examination of tissues and organs was performed at necropsy. Tissues and regions examined included all organs listed in the organ weight measurements above, as well as the following: thoracic aorta, bone marrow, cecum, colon, duodenum, oesophagus, eyes, femur, Harderian glands, head, ileum, jejunum, lungs, lymph nodes, pancreas, salivary glands, sciatic nerves, skeletal muscle, skin, spinal cord, sternum, stomach, trachea, bladder, and vagina.

The macroscopic examinations performed at the end of treatment or recovery periods revealed no ^13^C-fumarate-related findings. The incidence and distribution of all findings were considered unrelated to treatment.

- 1. Histopathology

As mentioned in the main text, minimal diffuse cortical vacuolation in the zona reticularis was observed in four of the ten female rats injected with 60 mg/kg of ^13^C-fumarate twice daily following the final injection on day 8 of the study. A similar observation was not seen in the corresponding dose group for male rats. Full recovery was noted following the two-week recovery period, and this observation was therefore considered to be non-adverse.

High basophil count was observed in the kidneys of roughly one quarter of the total population, whilst minor bruising to the injection site was observed in roughly half of the total population. These observations were noted at a similar incidence and distribution in animals that received the saline control, vehicle control or ^13^C-fumarate injections, and were therefore considered to be due to the injection procedure and unrelated to treatment.

# Validation of ^13^C-fumarate concentration and tolerability in healthy human volunteers

The maximum assessed ^13^C-fumarate dose concentration in the animal toxicology study was ~100 mM as discussed in Section 3. As part of a study into the tolerability of ^13^C-fumarate injection in healthy human volunteers, a maximum acceptable deviation in ^13^C-fumarate concentration of ±10% of the target was considered acceptable for injection into humans: e.g., for the highest target dose investigated of 80 mM, injection could only proceed if the assessed concentration after dissolution was between 72 and 88 mM inclusive as determined by mass spectrometry. The low (20 mM) dose consisted of 0.38 g [1,4-^13^C_2_,3-d_2_]fumarate mixed with 0.66 g DMSO in buffer and water for injection as described in Methods Section 4 of the main manuscript, with linearly-scaling quantities required for medium (40 mM) and high (80 mM) concentration doses. Since injections were performed at thermal polarization of ^13^C-fumarate, there was no requirement for inclusion of AH111501 radical in this study.

For the purposes of repeatability, included in Table S12 below is a summary of the various in vivo human experiments conducted, stating the target and actual recorded ^13^C-fumarate concentration values measured prior to injection.

| Participant | Target dose  mg/kg | Actual dose  mg/kg | Deviation  % | pH | Osmolarity |
| --- | --- | --- | --- | --- | --- |
| pt1 MFu01 | 0.96 | 0.9 | -6% | 8.09 | 626 |
| pt2 MFu03 | 0.96 | 0.92 | -4% | 7.78 | 327 |
| pt3 MFu04 | 0.96 | 0.96 | 0% | 7.23 | 684 |
| pt4 MFu02 | 1.92 | 2.05 | 7% | 7.86 | 377 |
| pt5 MFu07 | 1.92 | 1.99 | 4% | 8.02 | 369 |
| pt6 MFu08 | 1.92 | 2.26 | 18% | 7.84 | 368 |
| pt7 MFu05 | 3.84 | 3.84 | 0% | 8.09 | 460 |
| pt8 MFu09 | 3.84 | 3.84 | 0% | 8.29 | 454 |
| pt9 MFu06 | 3.84 | 3.84 | 0% | 8.2 | 432 |

**Table S12:** Repeatability of ^13^C-fumarate formulation to various target dose concentrations in the healthy human volunteer injections. 0.96 mg/kg corresponds to a concentration of 20 mM, 1.92 mg/kg to 40 mM, and 3.84 mg/kg to 80 mM.

Additional observations from the monitoring of subject blood hematology discussed in Figure 5 of the main text are presented in Figure S1 below for the following: hematocrit (Hct), hemoglobin concentration (Hb), erythrocyte count (RBC), total leucocyte count (WBC), red cell distribution width (RDW), mean cell hemoglobin (MCH), mean cell volume (MCV), mean platelet volume (MPV) and platelet count (Plt), as well as leucocyte concentrations for neutrophils (N), lymphocytes (L), eosinophils (E), basophils (B) and monocytes (M). Measurements of blood chemistry parameters in addition to those specified in the main text are as follows: alkaline phosphatase (ALP), alanine aminotransferase (ALT), aspartate aminotransferase (AST), total bilirubin (Bili), urea, creatinine (Creat), potassium (K) and albumin (Alb).


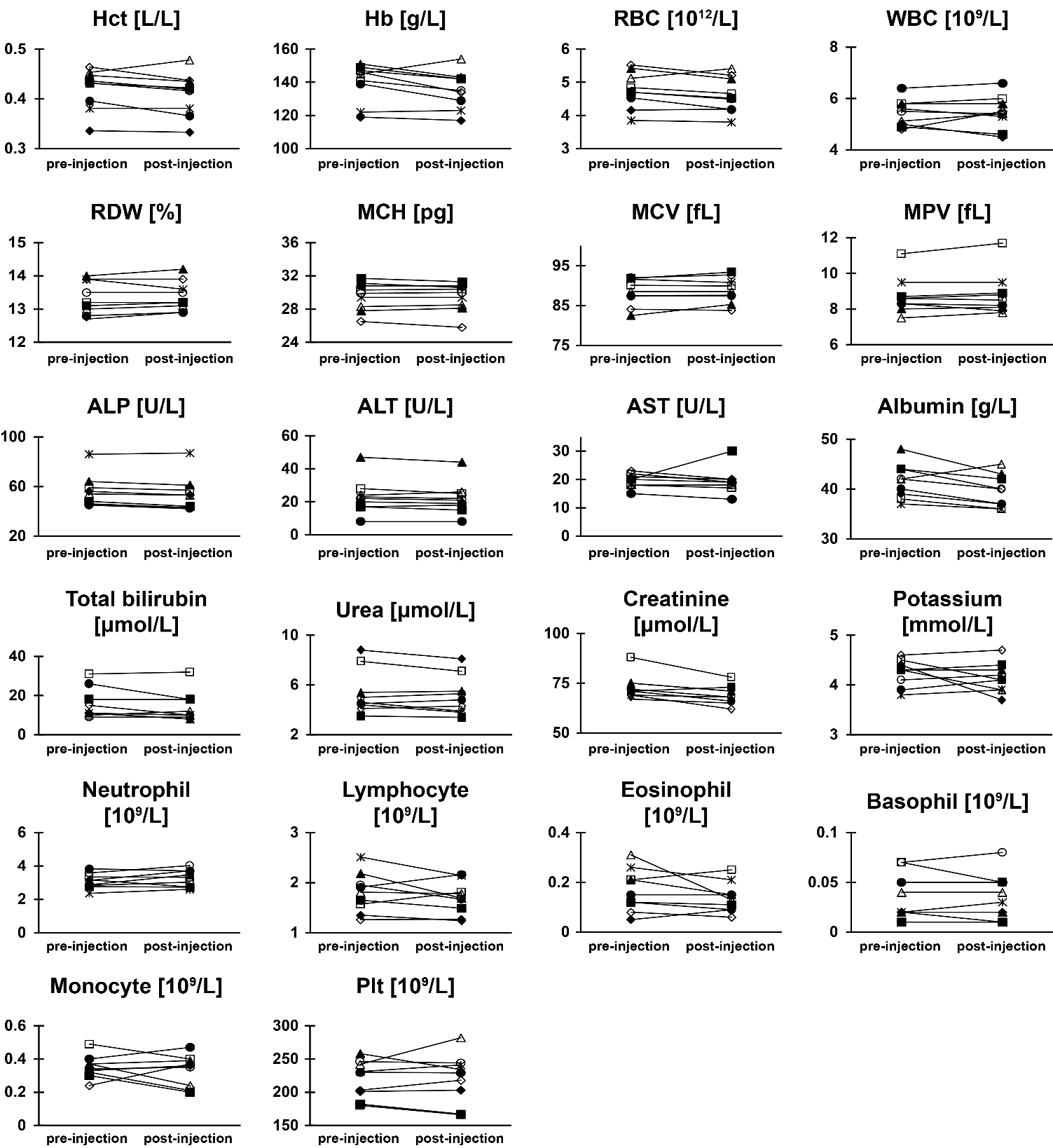


**Figure S1:** Peripheral blood hematology and blood biochemistry measurements obtained from the healthy human volunteer population before and after injection with ^13^C-fumarate at various dose levels and flow rates.

None of the observed parameters listed here were deemed to have either changed significantly or deviated beyond the normal ranges expected from healthy human volunteers as a consequence of ^13^C-fumarate injection.

# [1-^13^C]pyruvate microwave frequency and amplitude sweep

As the majority of ^13^C hyperpolarization studies to date have been performed using [1-^13^C]pyruvate as the target molecule, it is to be expected that DNP hardware is largely optimized for this molecule. Whilst we have demonstrated how to maximize polarization for [1,4-^13^C_2_,3-d_2_]fumarate in this study (see Figures 5a and 5b of the main manuscript), it would be simpler if ^13^C-pyruvate and ^13^C-fumarate hyperpolarization are similarly efficient in a single experimental configuration.

To assess this possibility, we conducted a comparable sweep of microwave frequency and attenuation for [1-^13^C]pyruvate. The results of these experiments are shown in Figure S2 below.


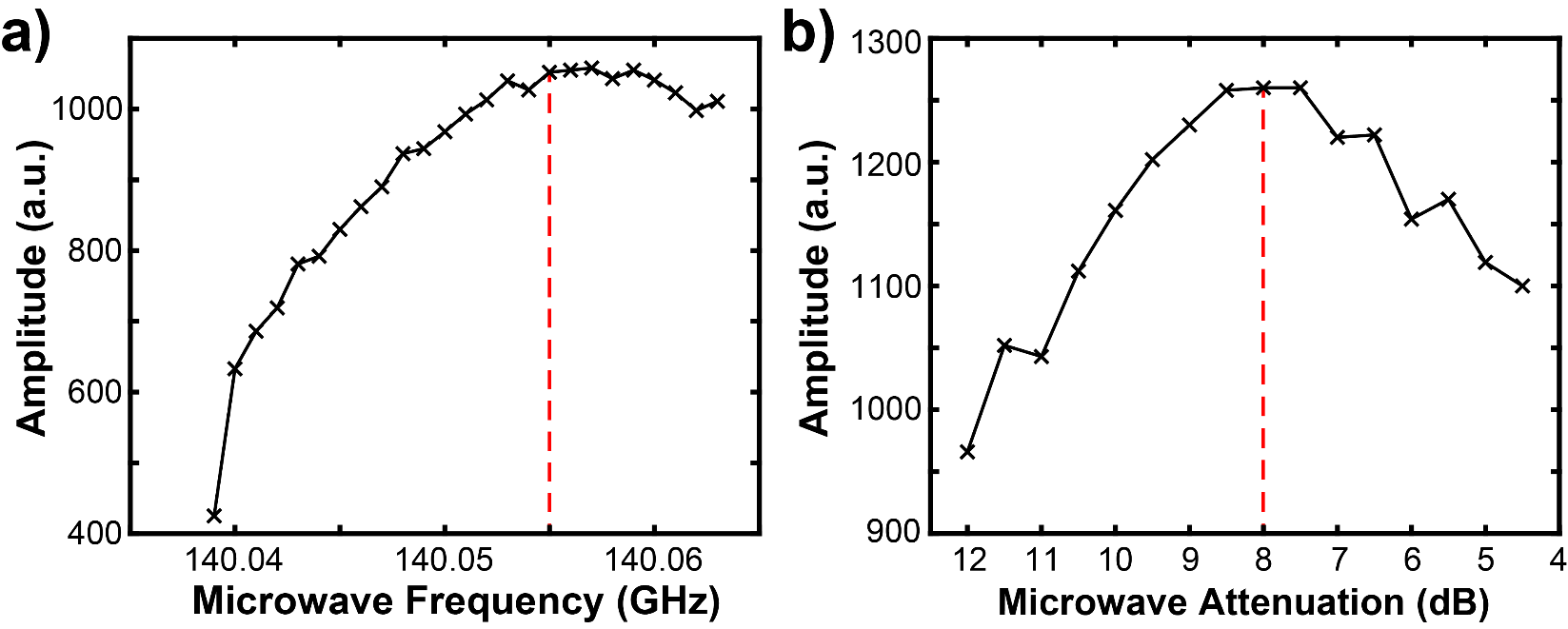


**Figure S2:** Optimisation of microwave a) frequency and b) attenuation for hyperpolarization of ^13^C-pyruvrate on the SPINlab hyperpolarizer. Vertical dashed red lines correspond to the values utilized in ^13^C-fumarate experiments described in the main manuscript.

The observation of near-maximal hyperpolarized ^13^C-pyruvate signal response at conditions identical to those optimized for ^13^C-fumarate in previous experiments highlights that both metabolites can be hyperpolarized efficiently under identical conditions due to the same radical being employed. This reduces the need for such optimization at sites where ^13^C-pyruvate hyperpolarization is already performed and removes a technical hurdle should co-polarization with sequential or joint administration of both ^13^C-pyruvate and ^13^C-fumarate be desirable in the future.

# Polarization build-up as a function of radical concentration

As part of the protocol development and optimization for ^13^C-fumarate hyperpolarization, a series of polarization build-up experiments were conducted with different concentrations of AH111501 radical: 17.5, 20, 25 and 30 mM. The final, optimized polarization build-up curve acquired at a radical concentration of 20 mM is shown in Figure 5d of the main text, and the corresponding polarization build-up curves for each of the other radical concentrations are shown here for comparison. Signal values were measured directly from the SPINlab hyperpolarizer without dissolution and sample ejection being performed.


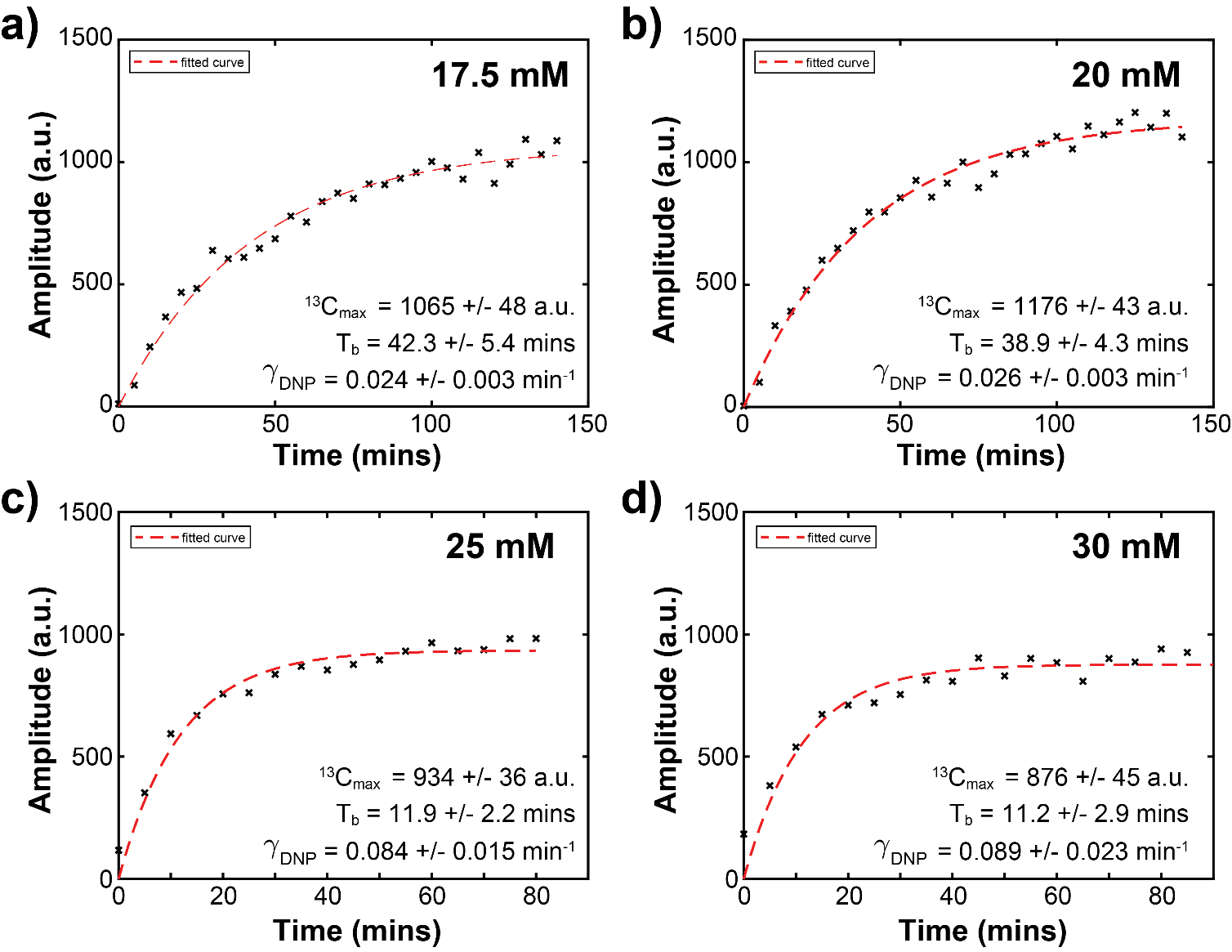


**Figure S3:** Polarization build-up curves acquired from otherwise identical (0.38 g fumarate, 0.66 g DMSO formulation) samples of ^13^C-fumarate as a function of AH111501 radical concentration utilized: a) 17.5 mM; b) 20 mM; c) 25 mM; d) 30 mM. Microwave frequency = 140.055 GHz, 8 dB attenuation, temperature = 0.8 K.

# Estimation of fumarate polarization in phantom studies

The fumarate polarization enhancement factor ε was calculated by comparing hyperpolarized and thermal ^13^C NMR peak integrals in phantom experiments using Equation S2

$\varepsilon=\frac{S_{13C}(HP)}{S_{13C}(thermal)}$ (S2)

The ^13^C-fumarate polarization at the point of signal acquisition was determined according to Equation S3

$P_{t}=\varepsilon\cdot tanh\left( \frac{\gamma\hbar B_{0}}{2k_{B}T} \right)$ (S3)

where *γ* represents the gyromagnetic ratio of the ^13^C nucleus in MHzT^-1^, *ħ* is the reduced Planck constant, *B_0_* is the magnetic field strength in Tesla, *T* is the temperature in Kelvin and *k_B_* is the Boltzmann constant. Fumarate polarization at the time of dissolution (*P_0_*) was then calculated using exponential fitting of the *T_1_* relaxation curve, performed using MATLAB (MathWorks, Natick MA) according to Equation S4

$P_{0}=P_{t}\cdot exp({t_{dis}}/{T_{1}})$ (S4)

where *t_dis_* represents the time delay in seconds between sample dissolution and signal acquisition, and *T_1_* is the calculated longitudinal relaxation time of the sample in seconds.

# Optical spectroscopic characterization of fumarate hydratase enzyme activity

Another component of the phantom imaging study was the validation of ^13^C-fumarate-to-malate conversion in the presence of different quantities of fumarate hydratase (FH) enzyme, as presented in Figure 6 of the main text. As further evidence for this process, a 1 mL sample of buffered and pH-neutralised ^13^C-fumarate was combined with 5 UmL^-1^ FH in a transparent glass cuvette, before performing optical absorption spectroscopy on the mixture at time intervals of one minute. A characteristic wavelength for fumarate of 290 nm was used. An enzyme concentration of 5 UmL^-1^ was chosen since it lay equidistant between the “low” and “high” FH concentrations utilised in the previous experiment (3.3 and 6.7 UmL^-1^, respectively).

Figure S4a shows the recorded optical spectrum from the ^13^C-fumarate and FH mixture at five-minute intervals, whilst Figure S4b displays the relative quantities of fumarate and malate in the mixture at each of these time points, as determined by performing an area-under-curve integration of the optical spectrum. Wavelengths limits for integration were chosen to be between 289 and 291 nm to reduce sensitivity to outliers.


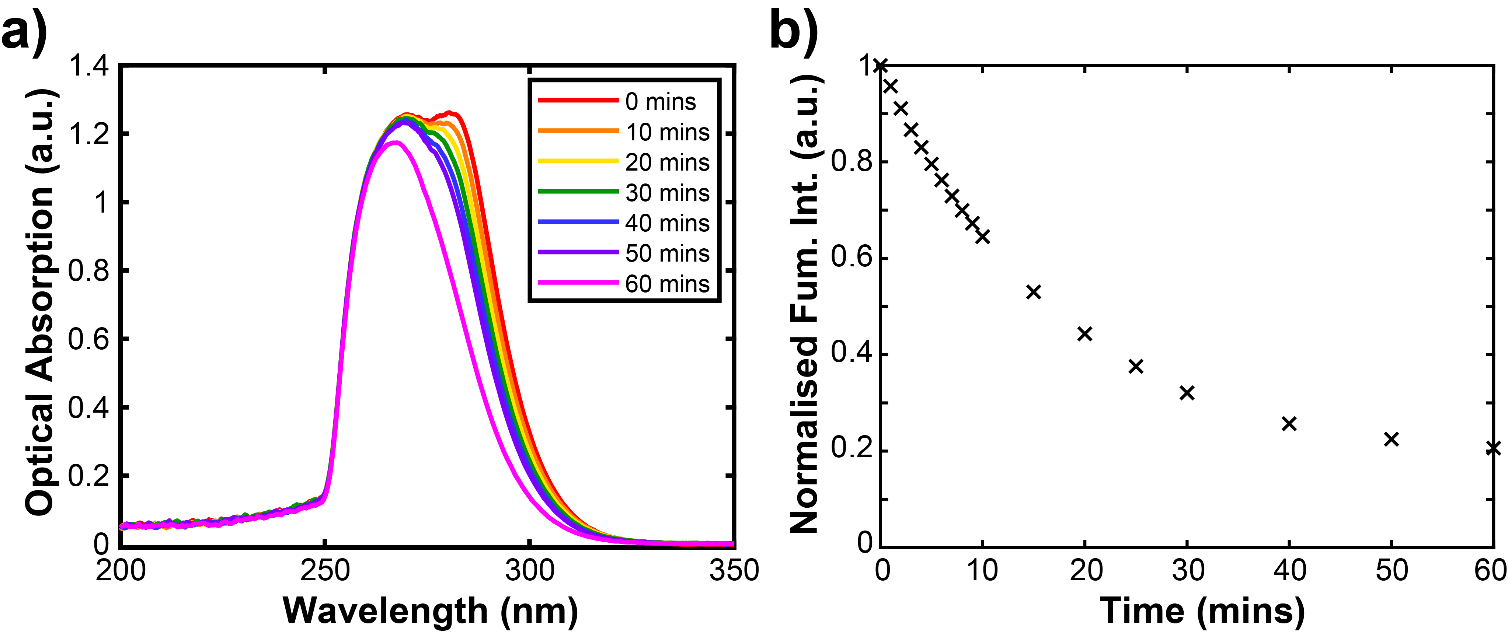


**Figure S4:** a) Optical spectra acquired from a 1 mL sample of ^13^C-fumarate as a function of time following addition of 5 UmL^-1^ FH; b) corresponding ^13^C-fumarate fraction of the total mixture at each time point as determined by area under curve integration of the optical spectra.

As evident from Figure S4a, the addition of FH to the the ^13^C-fumarate solution results in a depression of spectral intensity centered on the 290 nm fumarate wavelength following conversion to malate. At the enzyme concentration of 5 UmL-1 used in this experiment, it can be shown from Figure S4b that after 10 minutes, approximately 40% of the ^13^C-fumarate has undergone conversion.

# MRSI characterization of malate-to-fumarate ratio in vivo

As part of the in vivo imaging experiments to assess HP ^13^C-fumarate conversion to malate in a porcine model of ischemia reperfusion injury, 2D maps of malate-to-fumarate ratio were produced to highlight the increased rate of malate production in the ischemic kidney following necrosis. Example data presented in Figure 7 of the main manuscript was produced from a single animal (subject #6).

Of the six animals prepared for injection with ^13^C-fumarate, subsequent imaging was possible in three (inclusive of the example subject presented in Figure 7 of the main manuscript) — corresponding 2D maps of malate-to-fumarate ratio are presented in Figure S5a and Figure S5b. Of the first four animals, failures were due to either insufficient ^13^C-fumarate polarization for detection (subject #2) or an inability to perform sample ejection/dissolution (subjects #3 and #4). The high rate of equipment failure in early subjects is thought to be related to a logistical issue whereby two imaging sessions were scheduled to be performed per day. Sample preparation, injection and imaging for the final two animals was performed on separate days, with both experiments conducted successfully. A comparison of mean malate-to-fumarate ratio between the ischemic and healthy kidneys for the three animals where injection and subsequent imaging were performed successfully is presented in Figure S5c.

Lastly, Figure S5d numerically illustrates the comparison between malate-to-fumarate ratio in ischemic and healthy kidneys using a histogram plot by dividing Figure 8d of the main manuscript vertically between the two kidneys and quantifying all non-zero voxels on each side. Total number of non-zero voxels: ischemic kidney = 58, contralateral kidney = 82.


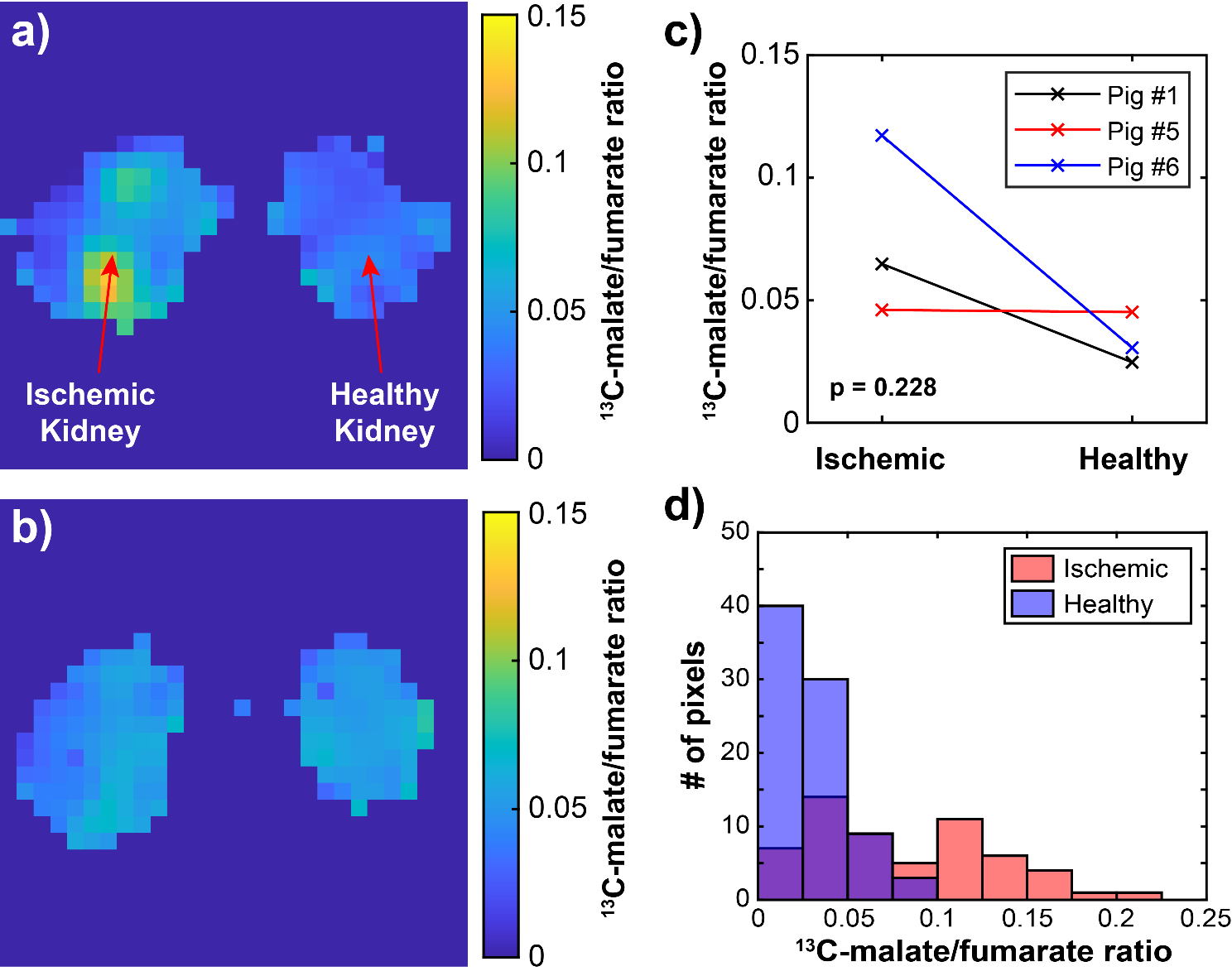


**Figure S5:** Spatial colormaps showing the measured malate-to-fumarate ratio in subjects #1 (a) and #5 (b); c) Difference in mean malate-to-fumarate ratio between ischemic and healthy kidneys in all animals; d) Histograms comparing malate-to-fumarate ratio in the ischemic and contralateral healthy kidneys for subject #6 (corresponding 2D MRSI colormap shown in Figure 7d of the main text).

Whilst little malate signal was detected in the ischemic kidney of subject #5 (Figure S5b), the 2D malate-to-fumarate ratio map for subject #1 (Figure S5a) did present an agreement with subject #6 in that elevated malate-to-fumarate ratio was observed in the ischemic kidney relative to the contralateral healthy control. This increase is quantified in Figure S5c: 113.0% for subject #1, 209% for subject #6 considering voxels within an ROI placed over the kidneys. Due to the low sample size, this increase was not found to be statistically significant as determined using a paired, two-tailed Student’s t-test (*p* = 0.228). The small sample size is a limitation of this work. The lack of elevated malate signal observed in one animal may be related to the unknown post-ischemia reperfusion duration (3-4 hours in this work) required to generate maximum necrosis in the large animal model used here, although this time-dependency has been investigated in rodent models[^1^](#_ENREF_1)^,^[^2^](#_ENREF_2). Further large animal studies with greater population sizes are warranted to fully understand how this information would be used in a clinical setting.

# References used in Supporting Information

1. Clatworthy MR, Kettunen MI, Hu D, et al. Magnetic resonance imaging with hyperpolarized [1,4-^13^C_2_]fumarate allows detection of early renal acute tubular necrosis. *Proceedings of the National Academy of Sciences.* 2012;109(33):13374-13379.

2. Nielsen PM, Eldirdiri A, Bertelsen LB, Jørgensen HS, Ardenkjaer-Larsen JH, Laustsen C. Fumarase activity: an *in vivo* and *in vitro* biomarker for acute kidney injury. *Scientific Reports.* 2017;7(1):40812.
